# Phosphorylation-state dependent intraneuronal sorting of Aβ differentially impairs autophagy and the endo-lysosomal system

**DOI:** 10.1080/15548627.2023.2252300

**Published:** 2023-08-29

**Authors:** Akshay Kapadia, Sandra Theil, Sabine Opitz, Nàdia Villacampa, Hannes Beckert, Susanne Schoch, Michael. T. Heneka, Sathish Kumar, Jochen Walter

**Affiliations:** a Molecular Cell Biology, Department of Neurology, University Hospital Bonn, Bonn, Germany; b Neuroinflammation Unit, German Center for Neurodegenerative Diseases e. V. (DZNE), Bonn, Germany; c Section for Translational Epilepsy Research, Department of Neuropathology, University Hospital Bonn, Bonn, Germany; d Microscopy core facility, University Hospital Bonn, Bonn, Germany; e Department of Neurodegenerative Disease and Geriatric Psychiatry, University Hospital Bonn, Bonn, Germany

**Keywords:** Alzheimer’s disease, autophagic flux, neurodegeneration, phosphorylated Aβ, post-translationally modified Aβ, vesicular trafficking

## Abstract

Progressive accumulation of amyloid-β (Aβ) aggregates in extracellular plaques is a characteristic hallmark of Alzheimer disease (AD). Aβ is also found in intraneuronal deposits and associated with alterations of the endo-lysosomal system and impairment of macroautophagy/autophagy. Here, we assessed the effect of Aβ phosphorylation on neuronal autophagy and the endo-lysosomal pathway. Analysis of APP-PSEN1dE9 transgenic mice revealed a phosphorylation-state dependent intraneuronal accumulation of Aβ species in endo-lysosomal and autophagy-related compartments. Cell biological studies further demonstrate a differential uptake and sorting of phosphorylated Aβ variants in cultured neurons, and phosphorylation-state specific effects of Aβ variants on neuronal autophagy and lysosomal function. While Aβ phosphorylated at serine residue 8 accumulated in autophagosomes, Aβ phosphorylated at serine residue 26 showed efficient transport to lysosomes. The selective sorting of phosphorylated Aβ species caused differential impairment of vesicular transport and lysosomal function associated with neurotoxicity. Thus, the relative occurrence of phosphorylated Aβ species and their intraneuronal accumulation could contribute to AD pathogenesis, and to the commonly observed aberrations of the vesicular transport system already at the early stages of the disease.

**Abbreviations:**

AD: Alzheimer disease; APP: amyloid beta precursor protein; ATG: autophagy related; Aβ: amyloid-β; CTSD: cathepsin D; DAPI: 4’,6-diamidino-2-phenylindole; EEA1: early endosome antigen 1; FA: formic acid; GFP: green fluorescent protein; LAMP2: lysosomal-associated membrane protein 2; MAP1LC3/LC3: microtubule-associated protein 1 light chain 3; MAP2: microtubule-associated protein 2; nmAβ: non-modified amyloid-β; npAβ: non-phosphorylated amyloid-β; pAβ: phosphorylated amyloid-β; p-Ser26Aβ: amyloid-β phosphorylated at serine residue 26; p-Ser8Aβ: amyloid-β phosphorylated at serine residue 8; RAB: RAB, member RAS oncogene family; RFP: red fluorescent protein; SQSTM1/p62: sequestome 1; YFP: yellow fluorescent protein.

## Introduction

Extracellular deposition of aggregated amyloid-β (Aβ) peptides is a hallmark of Alzheimer disease (AD) [1–3]. Aβ can also be detected in different vesicular compartments of neurons and intraneuronal Aβ is associated with impaired cellular homeostasis and neuronal loss [4–10]. Growing evidence suggests a close relation of intracellular Aβ and dysregulation of autophagy [7,8,11–13]. Autophagosomal vesicles are abundant in AD brains, featured by increased expression of autophagy related proteins in axonal dystrophies [14–16]. Electron microscopy studies revealed accumulation of autophagic vesicles associated with axonal swellings in proximity of Aβ plaques [13,17,18]. In turn, alterations in autophagic flux could impact Aβ clearance, thereby resulting in intraneuronal accumulation of Aβ species, and the impairment of synaptic function and neuronal metabolism [15,18–21]. Further reports also indicated that endocytosed Aβ disrupts endo-lysosomal compartments thereby contributing to neuronal death [7,8,19,21–26].

Aβ variants of various length have been identified that result from alternative processing of APP (amyloid beta precursor protein) and/or further cleavages of Aβ by several peptidases [27–31]. In addition, multiple post-translationally modified variants have been reported, including pyroglutamated and phosphorylated Aβ species [30,32,33]. We previously showed that extracellular Aβ can be phosphorylated by secreted and cell-surface localized protein kinases [34]. Phosphorylated Aβ (pAβ) peptides show differential aggregation characteristics and exert higher cytotoxicity compared to the non-phosphorylated variant (npAβ) [35]. pAβ Apecies also exhibit differential deposition in transgenic mouse models and human AD cases [34,35]. Here, we examined the relation of pAβ species with respect to neuronal autophagy and the endo-lysosomal pathway in a transgenic mouse model. We further employed *in vitro* cell culture models to examine the effects of distinct phosphorylated Aβ species (p-Ser8Aβ and p-Ser26Aβ) in comparison to the non-phosphorylated (np) Aβ peptide. Our results demonstrate that phosphorylation-state dependent intraneuronal accumulation and vesicular sorting of Aβ result in differential effects on autophagy and the endo-lysosomal system. Thus, the differential intraneuronal accumulation of phosphorylated Aβ species could contribute to dysfunction of autophagy and the endo-lysosomal system in the pathogenesis of AD.

## Results

### Differential intraneuronal distribution of phosphorylated Aβ species in autophagy and endo-lysosomal compartments in brains of APP transgenic mice

**Figure 1.**
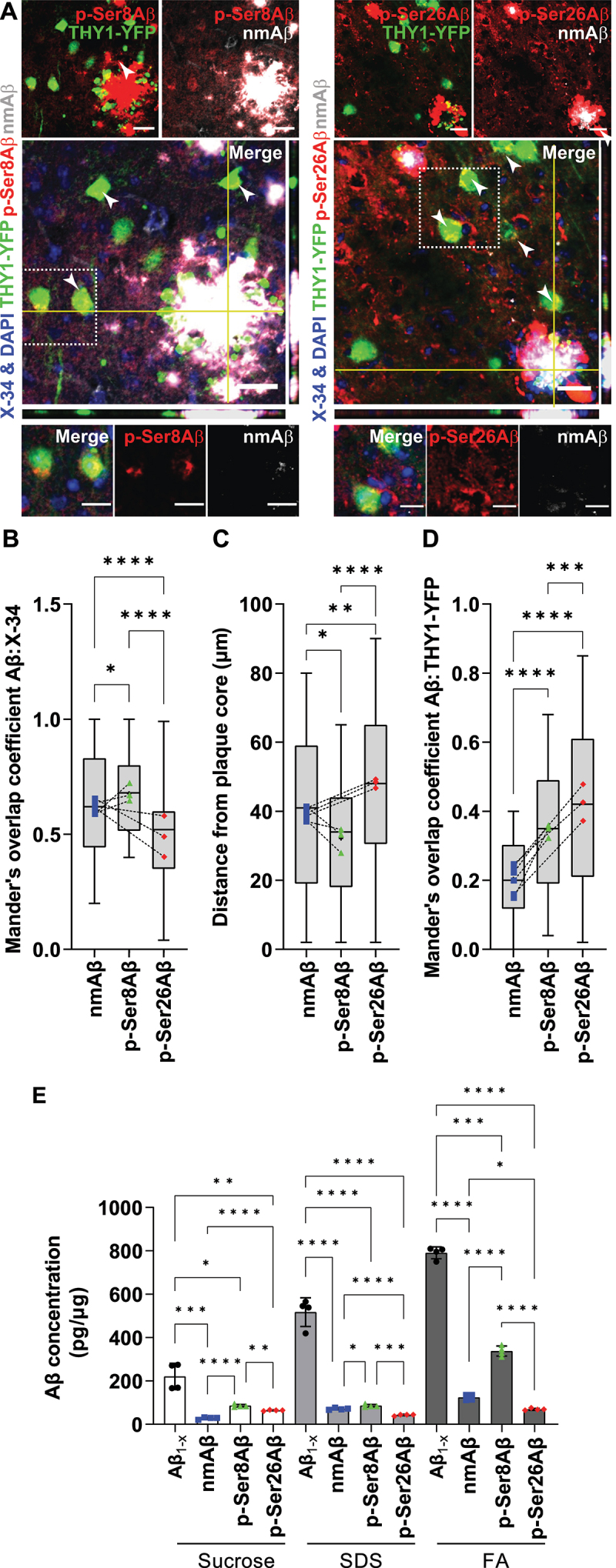
Differential intraneuronal deposition of pAβ species in APP-PSEN1dE9 transgenic mice. (A) Immunohistochemistry depicting differential intraneuronal and extracellular deposition of p-Ser8Aβ (stained with antibody 1E4E11, *red*) and p-Ser26Aβ (stained with antibody 5H11C10, *red*) compared to non-modified Aβ (nmAβ, stained with antibody 7H3D6, *gray*) in brain sections of APP-PSEN1dE9×THY1-YFP transgenic mouse cortex (7.4 m, female). Scale bar: 50 µm; zoomed panels, Scale bar: 10 µm. White arrowheads indicate colocalized punctate staining between red and gray channels in THY1-YFP-positive neurons. Source data have been provided in figure S8A. (B) Mander’s coefficient of overlap between nmAβ (*A, gray channel*; *B, blue data points*), p-Ser8Aβ (*A, red channel*; *B, green data points*) and p-Ser26Aβ (*A, red channel*; *B, red data points*) with X-34 (plaque core, *blue channel*). (C) Quantification of plaque association of nmAβ, p-Ser8Aβ and p-Ser26Aβ species. Distance from the plaque core was measured using Fiji ImageJ concentric circle processing module. (D) Mander’s coefficient of overlap between nmAβ (*blue data points*), p-Ser8Aβ (*green data points*) and p-Ser26Aβ (*red data points*) with respect to THY1-YFP-positive neurons (*green channel*) respectively. Box plot depicts the overall distribution of data, and each data point represents average values from an individual mouse, (A-B, *n* = ~100 cortical plaques), *N* = 3 transgenic mice. *p = 0.05; **p = 0.01; ***p = 0.001; ****p = 0.0001 (One-way ANOVA, GraphPad Prism). (E) Bar plots depicting the quantification of Aβ levels (pg/µg) in sucrose, SDS and formic acid (FA) soluble fractions by ELISA using phosphorylation-state specific antibodies, respectively (see the Materials and Methods section). Values represent mean ± S.D., from four different mouse brains per cohort, from two independent experiments, *n* = 4 mice, *N* = 2. *p = 0.05; **p = 0.01; ***p = 0.001; ****p = 0.0001 (two-way ANOVA, GraphPad Prism). Transgenic expression of human APP and accumulation of Aβ was verified by western immunoblotting (Figure S1). Additional information and further ELISA characterization of mouse brain lysates are provided in tables S1-S2, respectively.

We first detected Aβ variants phosphorylated at Ser8 (p-Ser8Aβ) or Ser26 (p-Ser26Aβ) as well as N-terminally non-modified Aβ (nmAβ) species in the APP-PSEN1dE9 transgenic mouse model that was crossed with THY1-YFP transgenic mice (APP-PSEN1dE9×THY1-YFP) to specifically label forebrain neurons (Table **S1**, Figure 1 and **S1**). Consistent with previous reports [34–38], the individual phosphorylated Aβ species (*red channels*, Figure 1A) show differential deposition when compared to nmAβ (*gray channels*, Figure 1A) or to total fibrillar Aβ detected by X-34 (*blue channels*, Figure 1A). p-Ser8Aβ and nmAβ species prominently localized within the core of extracellular plaques that contain X-34-stained fibrillar Aβ (Mander’s overlap coefficient, *R* – p-Ser8Aβ, 0.67 ± 0.17; nmAβ, 0.61 ± 0.22; Figure 1B). In contrast, colocalization of p-Ser26Aβ with X-34 was significantly lower (*R* = 0.48 ± 0.18), wherein, p-Ser26Aβ was also detected around the plaque core. The differential distribution of the Aβ phosphorylation-state variants within the plaque area was further confirmed by quantifying the distance of signals from the center of the X-34-stained plaque core (Figure 1C). p-Ser8Aβ is present within the compact core of the plaque, whereas p-Ser26Aβ is detected rather diffused from the core.

Mouse brains were also subjected to differential extraction and individual Aβ species detected by ELISA (Figure 1E, Table **S2**). Highest levels of total Aβ (Aβ_1-x_) were detected in the formic acid (FA) fraction that contains plaque associated fibrillar material. Consistent with the accumulation of p-Ser8Aβ in the plaque core, this species was also abundant in the FA fraction (Figure 1E, Table **S2**). Levels of p-Ser26Aβ were overall lower as compared to p-Ser8Aβ and did not show pronounced enrichment in the FA fraction, in line with the previously described effect of Ser26 phosphorylation to form soluble oligomeric assemblies, rather than fibrillar assemblies [35]. Aβ species were also detected in sucrose and SDS extracted fractions that predominantly contain extracellular soluble, and membrane/cell associated Aβ, respectively. While p-Ser8Aβ showed ~ 1.9-fold enrichment in the SDS versus the sucrose soluble fraction, p-Ser26Aβ species were enriched in the sucrose soluble fraction (1.5-fold) compared to the SDS fraction. Immunohistochemical analyses further revealed increased accumulation of both pAβ species within YFP-positive neurons (*R* – p-Ser26Aβ, 0.43 ± 0.24; p-Ser8Aβ, 0.35 ± 0.18; Figure 1D) as compared to nmAβ (*R* = 0.2 ± 0.11; Figure 1D). Together, these data indicate that the phosphorylation-state of Aβ not only affects its deposition in extracellular plaques, but also their accumulation inside of neurons.

**Figure 2.**
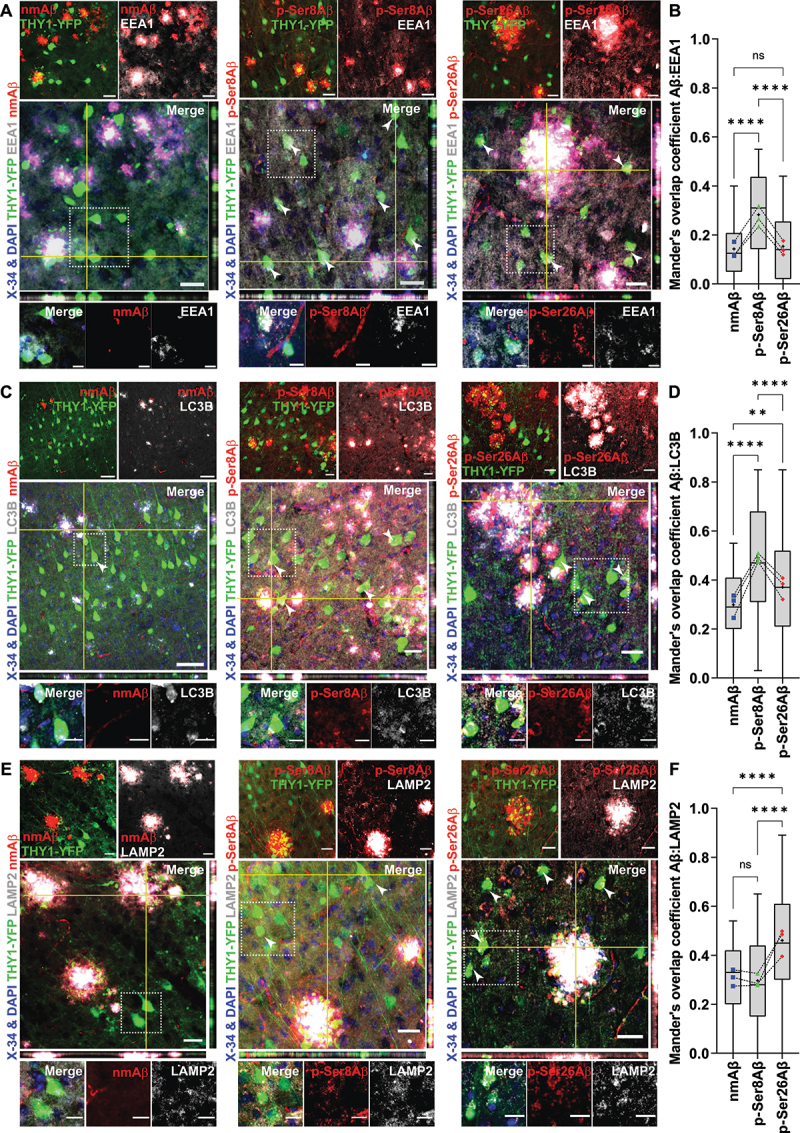
Differential intraneuronal colocalization of pAβ with vesicular marker proteins in APP-PSEN1dE9 transgenic mice. (A, C, E) Immunohistochemistry depicting differential intraneuronal colocalization of Aβ species in brain sections of APP-PSEN1dE9×THY1-YFP transgenic mouse cortex (7.3 m, female) stained with different phosphorylation-state specific Aβ antibodies (nmAβ-7H3D6, p-Ser8Aβ-1E4E11 and p-Ser26Aβ-5H11C10; *red channels respectively*) along with antibodies against EEA1 (*gray*, A); LC3 (*gray*, C) and LAMP2 (*gray*, E) and DAPI + X-34 (nuclei and plaque core, *blue*). Scale bar: 50 µm; zoomed panels, 10 µm. White arrowheads indicate colocalized punctate staining between red and gray channels in THY1-YFP-positive neurons. Source data have been provided in figure S8B-D. (B, D, F) Mander’s coefficient of overlap between red channels (npAβ, *blue*; p-Ser8Aβ, *green* and p-Ser26Aβ, *red data points*) with respect to gray channels (EEA1, B; LC3, D and LAMP2, F), quantified within THY1-YFP-positive neurons respectively. Box plot depicts the overall distribution of data, and each data point represents average values from an individual mouse, *N* = 3 transgenic mice. *ns* > 0.05; *p = 0.05; **p = 0.01; ***p = 0.001; ****p = 0.0001 (One-way ANOVA, GraphPad Prism). Additional staining and antibody control experiments are provided in figure S2.

We further examined localization of Aβ species with several marker proteins within distinct endo-lysosomal and autophagy related vesicles in YFP-positive neurons (Figure 2, **S2**, *Scheme –* Figure 8A). Intraneuronal pAβ species were detected in association with early endosomal (EEA1-positive) compartments (Figure 2A,B), wherein colocalization with EEA1 was significantly higher for p-Ser8Aβ (*R* = 0.27 ± 0.17) in comparison to p-Ser26Aβ (*R* = 0.15 ± 0.12) and nmAβ (*R* = 0.13 ± 0.10). Interestingly, p-Ser26Aβ showed highest colocalization with RAB7, a marker for late endosomes, followed by p-Ser8Aβ and nmAβ species (Figure **S2A-B**). pAβ species were also co-detected with LC3-positive autophagic compartments (Figure 2C,D) in neurons. As compared to nmAβ (*R* = 0.29 ± 0.13), p-Ser26Aβ (*R* = 0.37 ± 0.19) and in particular p-Ser8Aβ (*R* = 0.49 ± 0.20) showed significantly higher colocalization with LC3-positive vesicles. To analyze the association of Aβ species with lysosomes, we used LAMP2 (lysosomal-associated membrane protein 2; Figure 2E,F) and the lumenal protease CTSD (cathepsin D; Figure **S2C-D**) as specific markers. Intraneuronal p-Ser26Aβ showed significantly higher colocalization (*R* = 0.46 ± 0.22) with LAMP2 as compared to that of nmAβ (*R* = 0.30 ± 0.13) or p-Ser8Aβ (*R* = 0.29 ± 0.16), respectively. These observations were also comparable with the colocalization of pAβ and nmAβ variants with CTSD (Figure **S2C-D**). Together, these results indicate differential intraneuronal accumulation of Aβ phosphorylation-state variants in endo-lysosomal and autophagy related pathways in brains of APP transgenic mice.

### Phosphorylation-state dependent uptake and accumulation of Aβ by neurons

**Figure 3.**
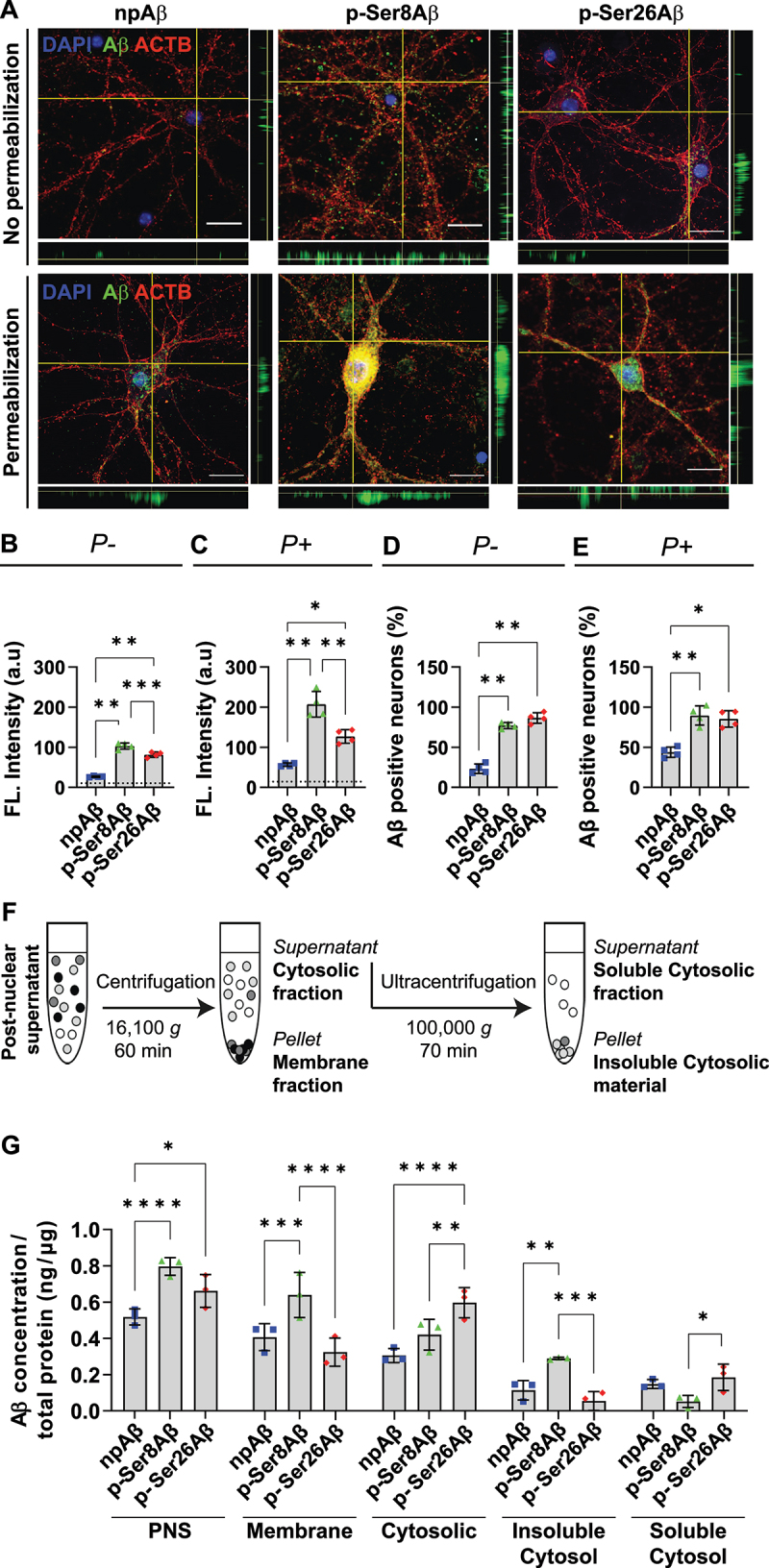
Phosphorylation-state dependent uptake and accumulation of Aβ in primary neurons. (A) Immunocytochemistry depicting Aβ accumulation in primary cortical neurons treated with the indicated Aβ variants (500 nM, 4 h). Cells were processed without permeabilization or with permeabilization to detect surface associated Aβ and internalized Aβ, respectively, by staining with anti-Aβ antibody 82E1 (*green*). Cells were co-stained with Alexa555-conjugated phalloidin (actin, *red*) and DAPI (nuclei, *blue*). Scale bar: 10 μm. (B, C) Bar plots depicting average values of absolute fluorescence intensities in the green channel (Aβ signals) in non-permeabilized cells (*P-*, B) and permeabilized cells (*P+*, C), analyzed by immunocytochemistry. Dotted line depicts the fluorescence signals in cells treated without Aβ (control). (D, E) bar plots depicting the quantification of neurons with surface bound Aβ (*P-*, E) and internalized Aβ (*P+*, D). Values represent mean ± S.D.; *n* = ~200 neurons, *N* = 4. *p = 0.05; **p = 0.01; ***p = 0.001; ****p = 0.0001 (One-way ANOVA, GraphPad Prism). (F) Workflow representation for fractionation of cellular protein based on differential centrifugation. (G) Quantification of absolute Aβ levels in different cellular fractions by ELISA (PNS, post nuclear supernatant) using anti-Aβ antibody 82E1. Values represent mean ± S.D.; *n* = 6, *N* = 3. *p = 0.05; **p = 0.01; ***p = 0.001; ****p = 0.0001 (two-way ANOVA, GraphPad Prism). Additional data and respective quantifications are shown in figure S3.

To specifically analyze the interaction, uptake, and intracellular accumulation of Aβ phosphorylation-state variants, we used cultured mouse primary cortical neurons that were exposed to synthetic npAβ, p-Ser8Aβ or p-Ser26Aβ peptides, respectively. Immunocytochemical detection of the different Aβ variants in neuronal cultures with or without plasma membrane permeabilization revealed significantly higher association of both phosphorylated Aβ species with the neuronal membrane surface, and increased accumulation in the cytoplasm (Figure 3A–E). Here, highest fluorescence intensities were detected for p-Ser8Aβ in both unpermeablized and permeabilized cells, indicating most efficient binding to neuronal membranes and intraneuronal accumulation, followed by p-Ser26Aβ and least for npAβ peptides (Figure 3B,C). Additional quantitative analyses further supported increased interaction of pAβ species with neurons. Here, the number of neurons with membrane bound Aβ (Figure 3D) and internalized Aβ (Figure 3E) is significantly higher after exposure to the pAβ species as compared to npAβ.

To complement these microscopic analyses, primary cortical neurons treated with different Aβ variants were fractionated by differential centrifugation to separate cellular membranes and cytosolic components (Figure 3F). Aβ levels in each fraction were then analyzed by ELISA (Figure 3G, **S3A-B**) and western immunoblotting (WB, Figure **S3E**). The post nuclear supernatant (PNS) that comprises cellular membranes and total cytosolic content, contained increased levels of both pAβ species, indicating increased cell association and/or accumulation as compared to npAβ (Figure 3G, **S3A-B**). Consistent with the increased fluorescence intensities for p-Ser8Aβ in microscopy analyses, this species also showed highest levels in the isolated membrane fraction (16,100 *g* pellet), while levels of npAβ and p-Ser26Aβ were significantly lower in this fraction. The total cytosolic fraction (supernatant after 16,100 *g* spin) showed highest levels for p-Ser26Aβ, followed by that of p-Ser8Aβ and npAβ. These data are consistent with increased binding and uptake of both phosphorylated Aβ species by neurons. Further separation of the cytosolic fraction by centrifugation at 100,000 *g* also revealed differential distribution of the phosphorylated Aβ species in soluble and insoluble fractions (Figure 3G, **S3A-B**). Levels of p-Ser8Aβ were significantly higher in the 100,000 *g* pellet as compared to npAβ and p-Ser26Aβ species, but significantly lower in the 100,000 *g* supernatant as compared to p-Ser26Aβ (the difference in levels of p-Ser8Aβ and npAβ in the 100,000 *g* supernatant was not statistically significant). Although the subcellular fractionation approach does not allow the identification of the different Aβ species in individual compartments, these data further support a differential subcellular distribution of phosphorylated Aβ in cultured neurons. WB analyses further revealed partial aggregation of Aβ species during the incubation with neurons, wherein p-Ser8Aβ forms more SDS resistant aggregates that npAβ or p-Ser26Aβ (Figure **S3E**).

We also analyzed the levels of Aβ in the treatment media by ELISA (Figure **S3C-D**). Levels of npAβ and p-Ser26Aβ were significantly decreased after 4 hours incubation with primary neurons (Figure **S3C**). Levels of p-Ser8Aβ show modest decrease but were not significantly different to the amount before incubation. Since, p-Ser8Aβ also showed the highest association with cultured neurons and intraneuronal accumulation, these data could indicate its higher metabolic stability. Indeed, we previously showed that phosphorylation of Aβ at Ser8 strongly decreases its degradation by Aβ degrading proteases [39], and this effect could also further contribute to the increased association of p-Ser8Aβ with neurons described above. These results indicate that phosphorylation of Aβ enhances membrane interaction and intraneuronal accumulation of the individual pAβ peptides. Alterations in aggregation states during these experiments could potentially contribute to the observed differences between the individual Aβ species and would reflect intrinsic effects caused by phosphorylation at the respective serine residues of Aβ. Very similar results described above for mouse primary neurons, were also obtained in another set of experiments with SH-SY5Y cells (Figure **S3B, D**).

### Site-specific phosphorylation of Aβ modulates its vesicular localization

**Figure 4.**
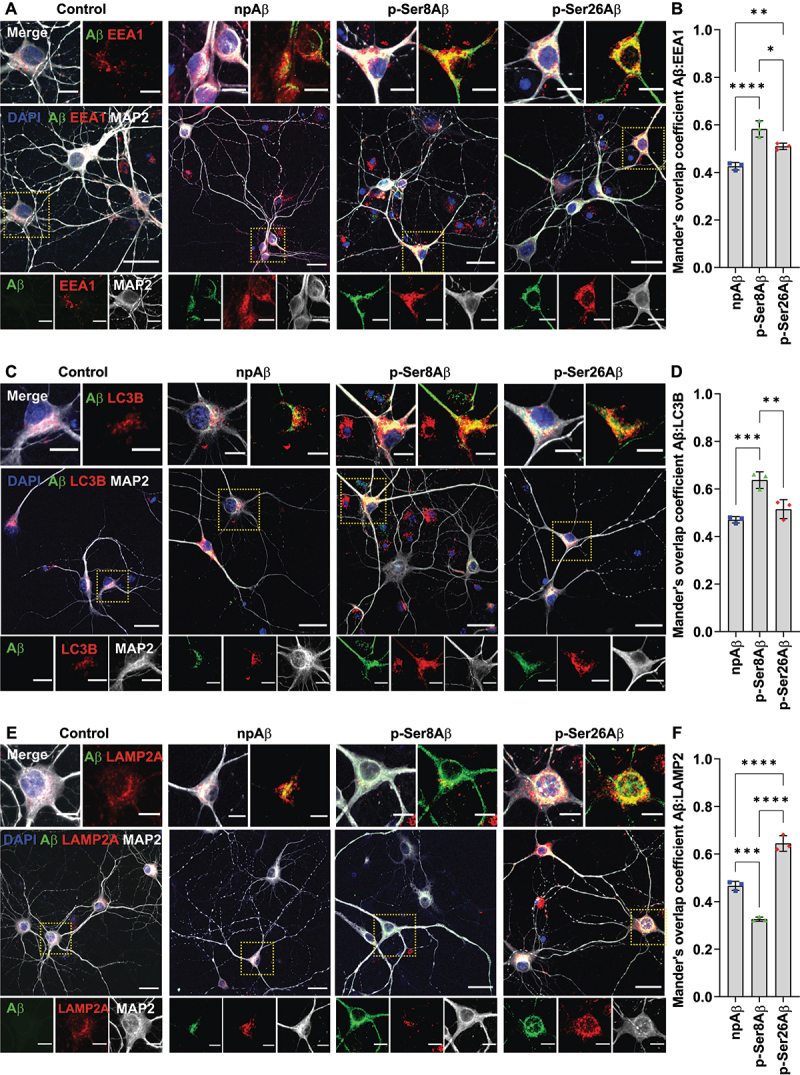
Phosphorylation-state specific intraneuronal sorting of Aβ to endo-lysosomal and autophagic compartments. (A, C, E) Primary cortical neurons were incubated without (control) or with the indicated Aβ variants (500 nM, 4 h) and co-stained with antibodies against the microtubule-associated protein 2 as neuronal markers (MAP2, *gray*), Aβ (82E1, *green*) and EEA1 (A, *red*), LC3 (C, *red*) or LAMP2 (E, *red*). Nuclei were stained with DAPI (*blue*). Scale bar: 10 µm. Dotted boxes indicate the regions zoomed in the merged panels (above) and individual channels (below). (B, D, F) Mander’s overlap coefficients between Aβ (*green channel*) with early endosomes, EEA1 (B); autophagic vesicles, LC3 (D) and lysosomes, LAMP2 (F) (respective red channels), analyzed by the Fiji ImageJ colocalization processing module. Values represent mean ± S.D.; *n* = 6, *N* = 3. * *p* = 0.05; ** *p* = 0.01; *** *p* = 0.001; **** *p* = 0.0001 (One-way ANOVA, GraphPad Prism). Additional data are shown in figure S4.

Once internalized into neurons via endocytosis, Aβ can be sorted to autophagy-related and endo-lysosomal compartments [8,25,40,41]. As our results with transgenic mouse brains indicated phosphorylation-state dependent distribution of intraneuronal Aβ in autophagic and endo-lysosomal compartments (Figure 2, **S2**), we next sought to determine the sorting of internalized Aβ species in cultured neurons (Figure 4, **S4**). Consistent with the observations with mouse brains, p-Ser8Aβ (*R* = 0.58 ± 0.18) showed significantly higher colocalization with EEA1-positive vesicles than p-Ser26Aβ (*R* = 0.51 ± 0.14) and npAβ (*R* = 0.43 ± 0.15) upon internalization into primary neurons (Figure 4A,B). Notably, the localization in RAB7-positive late endosomes of p-Ser8Aβ was lower (*R* = 0.33 ± 0.05) as compared to that of p-Ser26Aβ (*R* = 0.62 ± 0.15) and npAβ (*R* = 0.44 ± 0.10; Figure **S4A-B**). The internalized pAβ peptides also were differentially associated with autophagy and lysosome related compartments. p-Ser8Aβ colocalized with LC3-positive vesicles (*R* = 0.64 ± 0.21) to a higher extent (Figure 4C,D). Colocalization of p-Ser26Aβ and LC3 (*R* = 0.52 ± 0.09) also tended to be higher as compared to npAβ (*R* = 0.47 ± 0.09), although this difference was not statistically significant. Notably, p-Ser26Aβ showed a significantly higher colocalization with LAMP2 (*R* = 0.65 ± 0.13) and CTSD (*R* = 0.65 ± 0.10), as compared to npAβ (LAMP2, *R* = 0.45 ± 0.12; CTSD, *R* = 0.47 ± 0.09; Figure 4E,F and **S4C-D**). On the other hand, p-Ser8Aβ peptides showed the lowest colocalization with LAMP2 (*R* = 0.33 ± 0.08) and CTSD (*R* = 0.30 ± 0.06). Based on these observations it can be concluded that there is a differential routing of pAβ within endo-lysosomal and autophagy-related compartments, depending on the site of phosphorylation.

**Figure 5.**
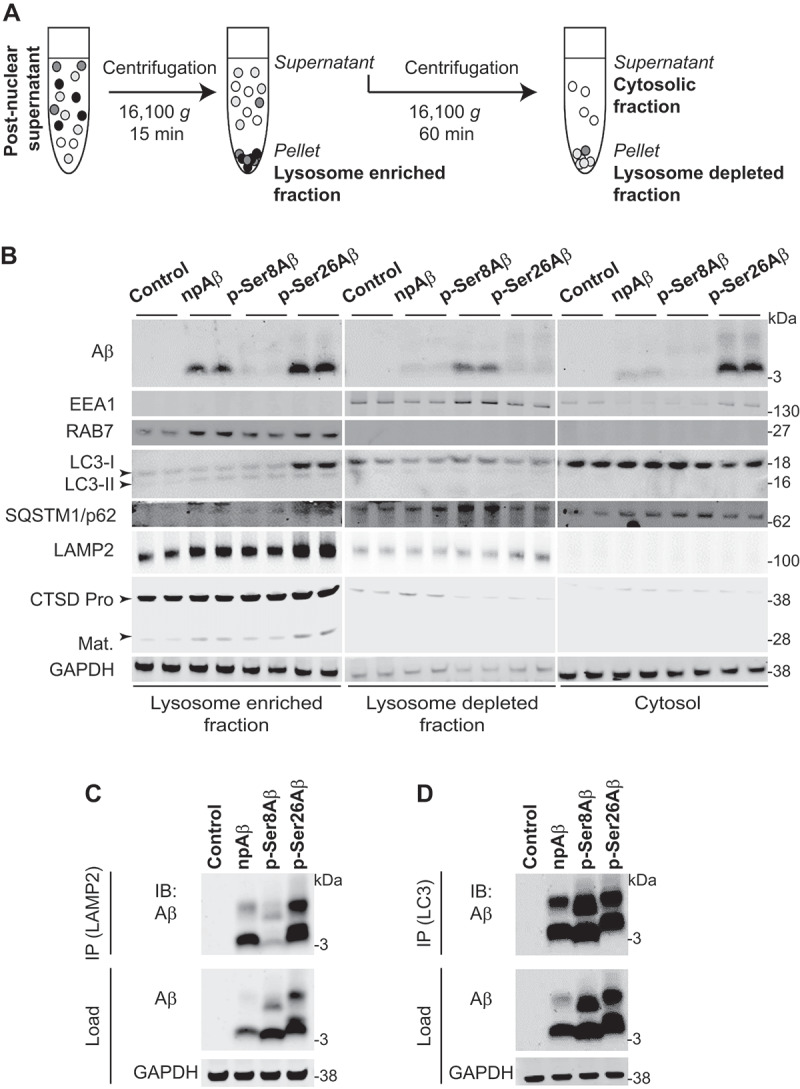
Phosphorylation-state dependent vesicular localization of Aβ. Primary cortical neurons treated with Aβ variants (500 nM, 4 h) were subjected to lysosome enrichment (A-B) or immunoisolation of LAMP2 (C) or LC3-positive compartments (D). (A) Workflow representation for lysosome enrichment based on differential centrifugation. (B) Immunoblot analysis of lysosome-enriched, lysosome-depleted, and cytosolic fractions depicting the differential separation of Aβ with the indicated endo-lysosomal and autophagy associated proteins. (C-D) Immunoisolation of intact lysosomes using LAMP2 (C) or autophagic vesicles (D) from cellular homogenate of primary cortical neurons treated with Aβ variants (500 nM, 4 h). Aβ in the isolated vesicles was detected with anti-Aβ antibody 82E1 via western immunoblotting. GAPDH and Aβ (82E1) were used as loading/starting controls. Results are representative of two independent experiments. IP, immunoprecipitation; IB, immunoblot. Respective quantifications and additional analyses are shown in figure S5.

To further assess the sorting of Aβ phosphorylation-state variants to lysosomal compartments, we used an additional cell fractionation protocol (Figure 5A). Here, LAMP2, CTSD, and RAB7 were preferentially detected in the 16,000 *g* pellet, indicating high enrichment of lysosomal/late endosomal compartments in this fraction (Figure 5B). Importantly, this fraction contains high levels of p-Ser26Aβ and moderate levels of npAβ, but only marginal levels of p-Ser8Aβ. Instead, p-Ser8Aβ is mainly found in the lysosome depleted fraction containing early endosomal (EEA1-positive) and autophagy associated SQSTM1/p62 (sequestome 1) and LC3-positive compartments (Figure 5B). p-Ser26Aβ and npAβ, but very little if any p-Ser8Aβ, is also found in the final supernatant that might contain other less dense vesicles, plasma membrane and cytosol. The differential distribution of the individual pAβ species was associated with alterations in the levels of individual marker proteins in the separated fractions, suggesting potential effects on the expression of these marker proteins (see below; Figure 8 and Table **S3**).

To further validate the differential sorting of the individual Aβ species, lysosomes from Aβ treated primary cortical neurons were immunoisolated using a LAMP2-specific antibody (Figure 5C). Again, p-Ser26Aβ and npAβ are strongly enriched in immunoisolated lysosomes as compared to p-Ser8Aβ (Figure **S5A-B**). A similar experimental set-up was used to immunoisolate autophagosomal components using a LC3 specific antibody (Figure 5D, **S5C-D**). Here, all Aβ variants were detected in immunoisolated LC3-positive autophagosomal compartments. In contrast to LAMP2 immunoisolated vesicles, the LC3 immunoisolated vesicles contain high levels of p-Ser8Aβ. Immunoprecipitation of total Aβ further supported the differential intraneuronal accumulation of Aβ peptides (p-Ser8Aβ > p-Ser26Aβ > npAβ, Figure **S5E-G**).

To further assess the subcellular localization of Aβ and potential effects on autophagy related vesicles, we performed scanning transmission electron microscopy (STEM) along with complementary immunocytochemistry on SH-SY5Y cells. Cells treated with the different Aβ species show accumulations of electron dense material within autophagic compartments, probably representing accumulated Aβ (Figure **S6A**). Notably, p-Ser8Aβ treated cells show accumulated autophagosomes still containing cargo material. On the other hand, p-Ser26Aβ as well as npAβ treated cells present with compartments containing less cargo material, indicative for autolysosomes wherein the cargo is already degraded. Parallel immunocytochemical analyses support a preferential accumulation of p-Ser8Aβ in LC3-positive autophagosomal structures (Figure **S6C, D**). In contrast, p-Ser26Aβ showed predominant colocalization with LAMP2 (Figure **S6E, F**). Quantification of autophagic compartments from STEM images (Figure **S6B**) suggest phosphorylation-state dependent effects of Aβ on autophagosome and/or lysosome accumulation. Taken together, these results depict phosphorylation-state specific trafficking and sorting of Aβ variants within autophagic and endo-lysosomal compartments, and also indicate potential effects on autophagic and/or lysosomal function.

### P-Ser8Aβ and p-Ser26Aβ differentially modulate autophagy

**Figure 6.**
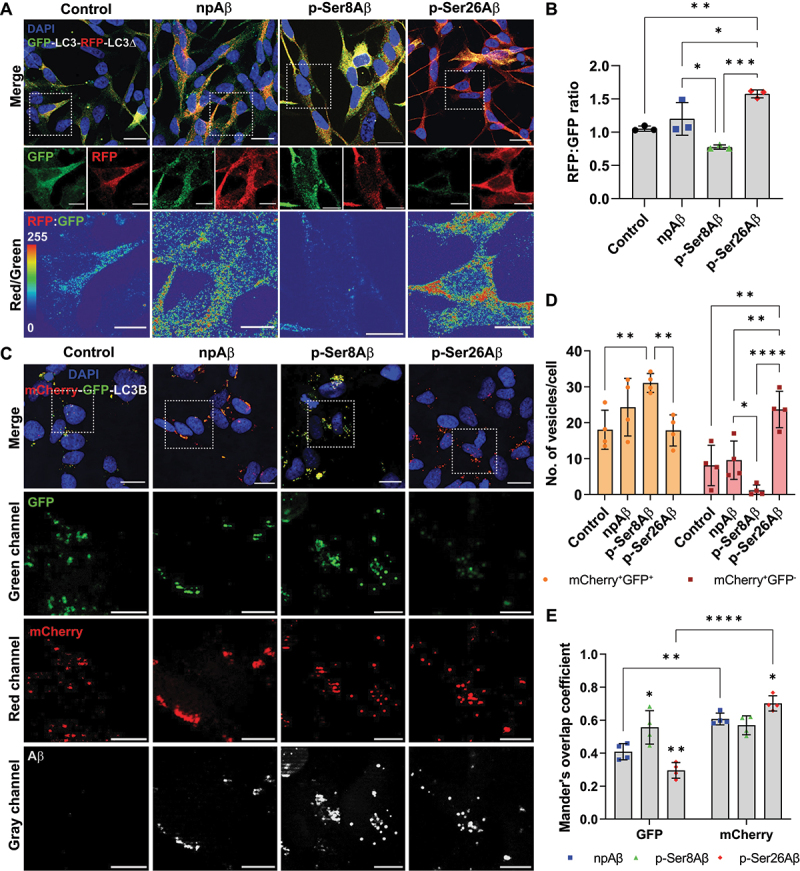
Phosphorylation-state specific effects of Aβ on autophagic vesicles. (A-E) Immunocytochemical analysis of SH-SY5Y cells expressing tandem reporter constructs GFP-LC3-RFP-LC3Δ (A-B) or mCherry-GFP-LC3B (C-E) upon treatment with different Aβ variants (1 µM, 24 h). Cells were co-stained with DAPI (nuclei, *blue*). Scale bar: 10 μm. Dotted boxes indicate the region zoomed in the panels below. (A) Merged channel images for GFP (*green*) and RFP (*red*) signals along with DAPI (nuclei, *blue*, upper row). Single channel images and RFP/GFP ratiometric images are shown in the middle and lower row, respectively. (B) Bar plot showing the ratio of RFP:GFP fluorescence intensities, respectively. Readings were normalized to cells incubated without Aβ (control). Values represent mean ± S.D.; *n* = 6, *N* = 3. *p = 0.05; **p = 0.01; ***p = 0.001; ****p = 0.0001 (One-way ANOVA, GraphPad Prism). (C) Merged channel images depict GFP (*green*) and mCherry (*red*) signals along with DAPI (nuclei, *blue*). Independent zoom regions below depict GFP (*green*), mCherry (*red*) and Aβ (*gray*) channels, respectively. (D) Quantification of the number of dual fluorescent (mCherry^+^ GFP^+^) and only mCherry-positive (mCherry^+^ GFP^−^) vesicles per cell representing differential autophagic flux. Values represent mean ± S.D.; *n* = 8, *N* = 4. (E) Mander’s overlap coefficient analysis of Aβ (*gray channel*) with GFP (*green channel*) and mCherry (*red channel*) positive vesicles analyzed using the Fiji ImageJ colocalization processing module. Values represent mean ± S.D.; *n* = 8, *N* = 4. *p = 0.05; **p = 0.01; ***p = 0.001; ****p = 0.0001 (Two-way ANOVA, GraphPad Prism). Additional analyses are shown in figure S7A-B.

To further investigate functional effects of phosphorylated and non-phosphorylated Aβ species on autophagosome formation and maturation (Figure 6A,B), we first used the tandem reporter construct GFP-LC3-RFP-LC3Δ [42]. Induction of autophagy results in the cleavage of the tandem reporter into two separate entities, GFP-LC3 that can be incorporated into phagophore membranes, and RFP-LC3Δ that serves as an internal control for expression of the construct. Fusion of autophagosomes with acidic lysosomes results in quenching of the fluorescence signals from the GFP. Thus, the ratio of RFP to GFP fluorescence intensity can provide a measure for autophagic flux. As shown in figure 6A-B, cell treatment with p-Ser26Aβ significantly increased the RFP:GFP ratio, indicating augmented autophagic flux as compared to control cells. Cell treatment with npAβ tended to increase, while treatment with p-Ser8Aβ tended to decrease the RFP:GFP ratio compared to that of control cells, but these effects were not statistically significant. The slightly lower RFP:GFP ratio in p-Ser8Aβ treated cells could indicate an increase in autophagic activation and/or inefficient fusion of GFP-LC3-positive vesicles with lysosomes, as compared to p-Ser26Aβ or npAβ treated cells.

We also used the alternative tandem reporter construct mCherry-GFP-LC3B [43,44] (Figure 6C–E and **S7A-B**). Here, the LC3 is fused with both fluorescent proteins, mCherry and GFP. Thus, LC3-positive compartments with neutral or weakly acidic pH show mCherry and GFP fluorescence simultaneously. Upon acidification of autophagosomes by fusion with lysosomes, the fluorescence of GFP, but not that of mCherry is quenched resulting in an increased ratio of mCherry:GFP fluorescence intensities [45]. The ratio of mCherry:GFP fluorescence intensities was strongly elevated in p-Ser26Aβ treated cells, but not significantly altered in p-Ser8Aβ and npAβ treated cells when compared to control cells (Figure **S7A**). The number of mCherry and GFP dual-fluorescent positive (mCherry^+^ GFP^+^) vesicles per cell is significantly higher in p-Ser8Aβ treated cells, compared to control and to p-Ser26Aβ treated cells (Figure 6D). However, the number of mCherry-positive/GFP-negative (mCherry^+^ GFP^−^) vesicles per cell is significantly least in p-Ser8Aβ treated cells, indicating inefficient transport to and/or impaired fusion with lysosomes. In contrast, cells treated with p-Ser26Aβ show a significantly higher number of mCherry^+^ GFP^−^ vesicles (Figure 6D), indicating increased autophagic flux and/or inefficient protein degradation in lysosomes. Mander’s overlap coefficient (Figure 6E) with GFP-positive vesicles is the highest for p-Ser8Aβ (*R* = 0.56 ± 0.10), followed by npAβ (*R* = 0.40 ± 0.05) and lowest for p-Ser26Aβ (*R* = 0.30 ± 0.05). On the other hand, p-Ser26Aβ shows highest colocalization with mCherry-positive vesicles (*R* = 0.70 ± 0.05) as compared to the other peptides (npAβ, *R* = 0.61 ± 0.04; p-Ser8Aβ, *R* = 0.57 ± 0.06). Analysis of the colocalization coefficients of the Aβ signals with GFP or mCherry channels depict the similar association of p-Ser8Aβ with both channels, supporting an accumulation of p-Ser8Aβ in non-acidic autophagic vesicles. In contrast, npAβ and in particular p-Ser26Aβ showed higher colocalization with mCherry than with GFP. These data suggest that site-specific phosphorylation of Aβ differently modulates autophagic flux with p-Ser8Aβ impairing autophagosome maturation and/or fusion to lysosomes, and npAβ and in particular p-Ser26Aβ rather affecting lysosomal function and/or acidification.

### Phosphorylation-state specific effects of Aβ on lysosomes

**Figure 7.**
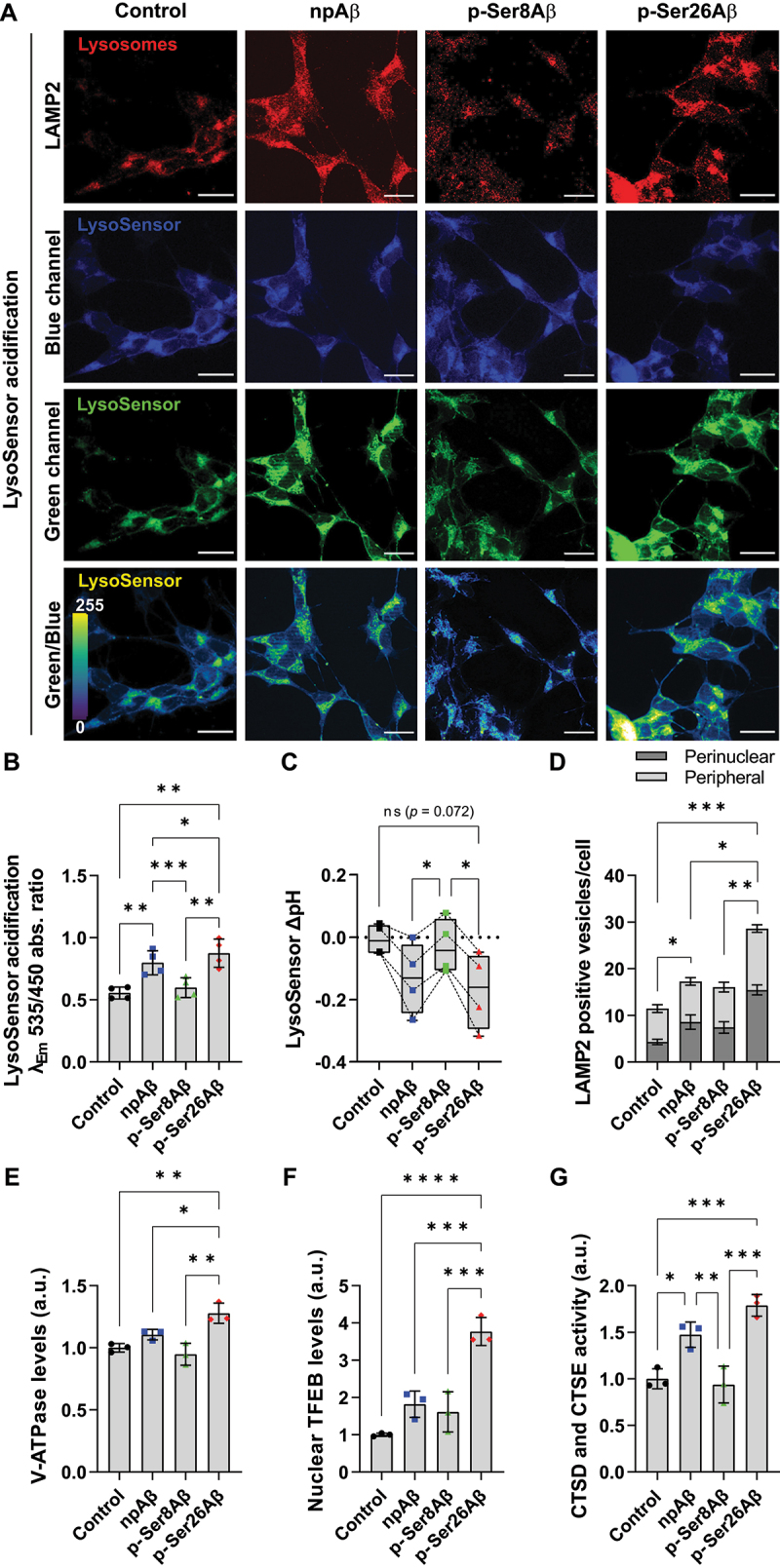
Differential effects of phosphorylated Aβ species on lysosomes. (A-C) SH-SY5Y cells loaded with LysoSensor-dextran complex were treated without or with the indicated Aβ variants (1 μM, 24 h). After incubation, cells were co-stained with anti-LAMP2 antibody to visualize lysosomes (*red*, A). Scale bar: 10 μm. Green/blue channel ratiometric images (*lower panel*, scale 0–255) indicate acidification of the LysoSensor. (B) Absolute ratios of fluorescence emission intensities (λ_Em_ 535 and 450 nm) measured at λ_Ex_ 380 nm were computed to determine the ratiometric response of LysoSensor acidification in SH-SY5Y cells treated with different Aβ variants (1 µM, 24 h). Values represent mean ± S.D.; *n* = 12, *N* = 4. *p = 0.05; **p = 0.01; ***p = 0.001; **** *p* = 0.0001 (One-way ANOVA, GraphPad Prism). (C) Change in LysoSensor pH (ΔpH) was quantified using the formula ΔpH = pH_final_ (control/Aβ, *t* = 24 h) − pH_initial_ (control, *t* = 0 h). Box plot depicts the overall distribution of data, and each data point represents average values from an independent experiment; *n* = 12, *N* = 4. *p = 0.05; **p = 0.01; ***p = 0.001; ****p = 0.0001 (repeated measures one-way ANOVA, GraphPad Prism). (D) Quantification of the number of LAMP2-positive compartments within the perinuclear (<2 µm from nucleus) or peripheral space (>2 µm from nucleus until cell periphery) per cell. Values represent mean ± S.E.M.; *n* = 12, *N* = 3. *p = 0.05; **p = 0.01; ***p = 0.001; ****p = 0.0001 (One-way ANOVA, GraphPad Prism). (E-F) Quantification of western immunoblot data from SH-SY5Y cells upon treatment without (control) or with the indicated Aβ variants (1 μM, 24 h), for the ATP6V0A1 subunit of the V-ATPase in the PNS fraction (E) and TFEB (transcription factor EB) in the nuclear material (F). Values were normalized to control cells and are presented as mean ± S.D.; *n* = 6, *N* = 3. (G) SH-SY5Y cells treated with Aβ variants (1 µM, 24 h) or without (control) were examined for CTSD and CTSE activity (per µg of total cellular protein) using a CTSD and CTSE cleavable fluorogenic substrate. Readings were normalized to control cells; values represent mean ± S.D.; *n* = 6, *N* = 3. *p = 0.05; **p = 0.01; ***p = 0.001; ****p = 0.0001 (One-way ANOVA, GraphPad Prism). Time-dependent lysosomal acidification analysis and additional controls are shown in figure S7C-F.

The results with both reporter constructs indicated differential effects of the Aβ phosphorylation-state variants on autophagic flux or on the quenching of GFP fluorescence. Thus, we next assessed their effects on lysosomes using a LysoSensor-dextran ratiometric probe by microscopy (Figure 7A) or time-dependent fluorescence analysis in multi-well plates (Figure 7B,C and **S7C-E**). The fluorescence emission of the probe changes from blue to green channel (*red shifted*) upon vesicle acidification, and the ratio of green to blue fluorescence intensity provides a measure for lysosomal acidification (Figure 7A, *lowermost panel*). Cell treatment with p-Ser26Aβ and to a lesser extent npAβ lead to increased signals indicating acidification of the LysoSensor, while p-Ser8Aβ had no significant effect (Figure 7B, **S7C-D**). Further quantitative analysis of the LysoSensor response (ΔpH, Figure 7C and **S7E**) indicated that p-Ser26Aβ only slightly decreased the pH by 0.17 ± 0.12 units within 24 h of incubation. A similarly small decrease in the pH by 0.13 ± 0.11 units is also seen in npAβ treated cells. Contrastingly, p-Ser8Aβ did not show any change in pH response of the LysoSensor (Figure 7C and **S7E**). Further, p-Ser26Aβ and to a lower extent npAβ significantly increased the number of LAMP2-positive vesicles (Figure 7A, *red channel*). Distribution analysis (Figure 7D) revealed an increase in perinuclear lysosome positioning for cells treated with the individual Aβ variants. The ratio of perinuclear to peripheral lysosomes was highest for p-Ser26Aβ (1.17 ± 0.05), followed by npAβ (0.99 ± 0.16) and least for p-Ser8Aβ, 0.88 ± 0.19), in comparison to control conditions (0.61 ± 0.12), respectively. Moreover, the number of LAMP2-positive vesicles was significantly increased in npAβ and particularly in p-Ser26Aβ treated cells (Figure 7D). Together, these data indicate differential effects of Aβ phosphorylation-state variants on lysosomal acidification and biogenesis. Indeed, p-Ser26Aβ significantly increased levels of both, nuclear transcription factor EB (TFEB) and intracellular levels of the ATP6V0A1 subunit of the V-ATPase, two major regulatory proteins of lysosomal biogenesis and lysosomal acidification, respectively (Figure 7E,F). The increase in the number of intracellularly accumulated lysosomes in p-Ser26Aβ treated cells was associated with increased activity of lysosomal CTSD and CTSE, while p-Ser8Aβ had no significant effect as compared to control cells (Figure 7G and **S7F**).

### Phosphorylated Aβ variants differentially affect autophagic and endocytic pathways, and exert higher cytotoxicity

**Figure 8.**
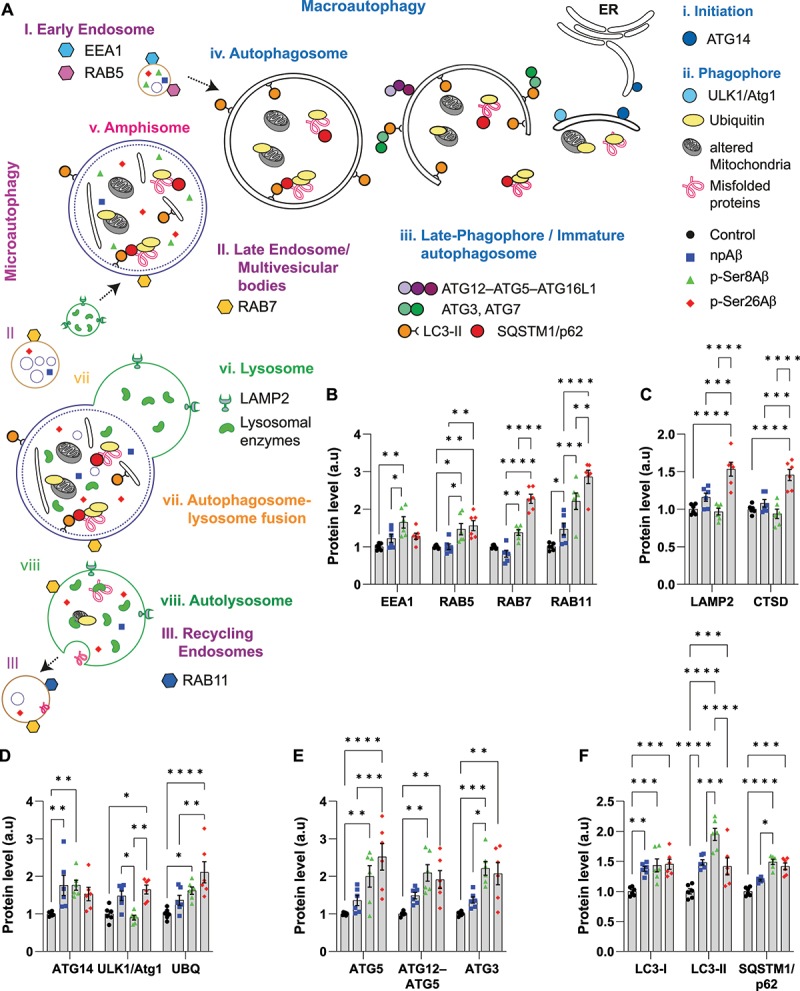
Dysregulation of autophagy-endo-lysosomal pathway by different Aβ species. (A) Schematic depicting major components in the distinct phases of the autophagy-endo-lysosomal pathway. Respective symbols indicate the localization of p-Ser8Aβ (*green triangles*) and p-Ser26Aβ (*red diamonds*) in comparison to npAβ (*blue squares*) peptide. (B-F) Quantification of western immunoblot data from SH-SY5Y cells for proteins involved in the individual phases of autophagy and endo-lysosomal function upon treatment without (control) or with the indicated Aβ variants (1 μM, 24 h). Values represent mean ± S.E.M.; *n* = 6, *N* = 3. Representative data are provided in figure S8E.

The results described so far indicate a phosphorylation-state dependent sorting and functional effects of Aβ on different autophagic and endo-lysosomal compartments. Thus, we next analyzed the effects of the different Aβ species on individual proteins functionally involved in macroautophagy and endocytic trafficking (Figure 8A, *scheme*) by quantitative western immunoblotting. The combined results obtained with SH-SY5Y cells and primary neurons are shown in figure 8B–F and Table **S3**, respectively.

Early endosomal proteins EEA1 and RAB5 were increased by cell treatment with p-Ser8Aβ. p-Ser26Aβ also increased RAB5 but had no significant effect on EEA1. p-Ser26Aβ had the strongest effects on RAB7 and RAB11 that mediate trafficking and function of late and recycling endosomal compartments, respectively. npAβ had no significant (EEA1, RAB5, RAB7) or smallest effect (RAB11) on levels of these endosomal proteins (Figure 8B). Notably, lysosomal proteins LAMP2 and CTSD were selectively increased by p-Ser26Aβ (Figure 8C).

It is interesting to note that all Aβ species caused elevated levels of ATG14 (Figure 8D) that is required for the initiation of autophagy [46]. npAβ and p-Ser26Aβ, but not p-Ser8Aβ, also increased the ATG1-ULK1 kinase complex that is also involved autophagosome-lysosome fusion [47,48]. Analysis of additional proteins involved in phagophore and autophagosome formation revealed that in particular the treatment with the two phosphorylated Aβ species leads to an increase in ATG5, and the ATG12–ATG5 complex (Figure 8E). Similarly, both phosphorylated Aβ species, but not npAβ, significantly increased levels of ATG3.

Cell treatment with the individual Aβ variants, in particular with p-Ser8Aβ, resulted in significantly increased levels of LC3-I and II as compared to control cells (Figure 8F). This indicates an increase in autophagosome formation or impaired autophagic flux in cells treated with p-Ser8Aβ as compared to p-Ser26Aβ and npAβ treated cells. Treatment with both pAβ variants significantly increased the levels of SQSTM1/p62, a receptor for autophagic substrates that is itself degraded during completion of autophagy (Figure 8F). Thus, the increased LC3-II levels and accumulation of SQSTM1/p62 upon treatment with p-Ser8Aβ could also indicate impairment of autophagic flux caused by decreased fusion of autophagosomes with lysosomes [45]. More importantly, both phosphorylated Aβ species induced a significant accumulation of ubiquitinylated proteins (Figure 8D), indicating impaired protein turnover. Overall, similar data were obtained with primary cortical neurons (Table **S3**). Together, the comprehensive analysis of autophagy and endo-lysosomal pathway related proteins revealed complex and differential effects of Aβ phosphorylation-state variants at specific phases during autophagy that result in impaired protein degradation. Further analyses indicated that the differential effects of the Aβ phosphorylation-state variants on the autophagy related and endo-lysosomal system were associated with differential effects on cell viability. p-Ser26Aβ exerted highest neurotoxicity followed by p-Ser8Aβ and npAβ (Figure **S7G-H**).

## Discussion

The present study with transgenic mouse brains and neuronal cell culture models revealed the phosphorylation-state specific intraneuronal sorting and localization of Aβ in distinct autophagic and endo-lysosomal compartments. The differential sorting of Aβ phosphorylation-state variants was associated with distinct effects on autophagic and endo-lysosomal functions, and neuronal viability.

Besides the deposition of Aβ in extracellular plaques, intraneuronal Aβ accumulation could also play important roles in neuronal dysfunction during the pathogenesis of AD [9,15,49,50]. Here, we identified differential localization of non-modified and phosphorylated Aβ species. As shown previously, p-Ser8Aβ was prominent in the fibrillar core of extracellular Aβ plaques [34,36,37,51], while p-Ser26Aβ is more diffusely associated with extracellular plaques [35,37]. More importantly, using a specific mouse model with YFP-labeled forebrain neurons, both phosphorylated Aβ species showed differential intraneuronal localization in autophagic and endo-lysosomal compartments. While p-Ser8Aβ showed higher colocalization in LC3-positive autophagic vesicles, p-Ser26Aβ was enriched in late endosomal and lysosomal compartments, including autolysosomes. Intraneuronal Aβ was described in different mouse models [6,7,10,24,52,53], and thus, it will be interesting to also analyze the vesicular distribution of phosphorylated Aβ species in additional APP transgenic mouse models in the future. It also remains to be determined whether the differential accumulation of phosphorylated Aβ species in brain neurons *in vivo* results from neuronal uptake of already phosphorylated extracellular Aβ species or from intracellular generation and phosphorylation of Aβ. Previous studies indicated that APP and APP C-terminal fragments can be targeted to endo-lysosomal and autophagic compartments and then processed to intravesicular Aβ [10,14,25,44,54–58]. Whether the pool of Aβ generated inside of vesicles can undergo phosphorylation remains to be investigated. In human AD brains, protein kinase CSNK1 isoforms were found in GVD compartments [59] that also contain p-Ser26Aβ, and *in vitro* experiments demonstrated phosphorylation of Aβ by CSNK1 activity [35]. In addition, intravesicular and secreted protein kinases have been identified that phosphorylate secreted and cell surface localized proteins [60,61], including APP [62,63]. The present study focussed on the uptake and intracellular sorting of phosphorylated Aβ by neurons, and the functional implications on autophagy and the endo-lysosomal system.

Neuronal autophagy and the endo-lysosomal pathway play an important role in the intracellular trafficking and accumulation of Aβ [15,18,21,40,41,56,57,64,65]. Notably, intraneuronal Aβ accumulation correlates well with neuronal dysfunction and neurodegeneration, and could contribute to cellular Aβ propagation and Aβ plaque formation [15,20,40,66,67]. Our studies with cultured neurons demonstrated that both phosphorylated Aβ species showed higher association with neuronal membranes as compared to npAβ. Once internalized into early endosomal compartments, p-Ser26Aβ is efficiently targeted to late endosomal and lysosomal compartments, while p-Ser8Aβ predominantly accumulates within early endosomal and autophagosomal structures. Thus, the phosphorylation-state could modulate the accumulation of Aβ in distinct intraneuronal vesicular compartments. However, the exact intracellular routes and the involvement of distinct autophagy dependent and independent vesicular compartments in the sorting of phosphorylated Aβ species need to be characterized in more detail, e.g., by live cell imaging with fluorescent Aβ species and high-resolution microscopy techniques. For example, it is possible that fractions of internalized Aβ variants, in particular of p-Ser26Aβ, could be transported in the endocytic pathway to lysosomes without initial involvement of autophagic vesicles. Such analyses could also provide further insight into the exact effects of distinct Aβ species in vesicular trafficking. Since recent evidence supports a critical role of intraneuronal Aβ accumulation in the formation of extracellular plaques [10,12,15,24,29,55], it will also be interesting to dissect the relative contribution of intraneuronal phosphorylated Aβ species to the generation of extracellular plaque pathology in the future.

Our results indicate differential effects of individual phosphorylated Aβ species on the autophagic process and lysosomal function. The accumulation of internalized p-Ser8Aβ in endosomal or autophagosomal vesicles could impair efficient delivery of cargo or vesicular fusion with lysosomes as suggested from results with the mCherry-GFP-LC3 probe. Here, p-Ser8Aβ increased the number mCherry^+^ GFP^+^ vesicles but decreased the number of mCherry^+^ GFP^−^ vesicles. This is indicative for increased formation and/or accumulation of autophagic vesicles, but decreased delivery to acidic lysosomal compartments. In contrast, p-Ser26Aβ had no significant effect on the number of mCherry^+^ GFP^+^ vesicles, but strongly increased the number of mCherry^+^ GFP^−^ vesicles, indicating efficient delivery of the construct to lysosomes during autophagic flux. Interestingly, our data also indicates that particularly p-Ser26Aβ, and to a lesser extent probably also npAβ and p-Ser8Aβ, could promote lysosomal biogenesis and augment intracellular accumulation of lysosomes. However, it remains to be determined in more detail whether the minor alterations in pH upon incubation with p-Ser26Aβ observed in this study translate to functional alterations or rather indicate a response to Aβ induced impairment of lysosomal function. Indeed, the accumulation of ubiquitinylated proteins and SQSTM1/p62 suggests lysosomal failure upon persistent exposure to p-Ser26Aβ associated with impairment of neuronal viability. Although p-Ser8Aβ was not efficiently targeted to lysosomes, this species also exerted comparable neurotoxicity. Here, the accumulation of p-Ser8Aβ in autophagic vesicles might impair vesicular transport and fusion with lysosomes, as supported by the accumulation of LC3-positive vesicles, and autophagic protein substrates, including SQSTM1/p62 and ubiquitinylated proteins. However, additional modes of Aβ induced neurotoxicity like perturbation of plasma membrane integrity or rupture of vesicular membranes and Ca^2+^ dysregulation could also contribute to the impairment of neuronal viability [8,15,68–70].

Phosphorylation affects aggregation characteristics of Aβ. While phosphorylation at Ser8 promotes formation of fibrillar assemblies, phosphorylation at Ser26 stabilizes oligomeric assemblies [35,71]. Although we added monomeric Aβ to neuronal cultures, western blot analysis indicated formation of Aβ aggregates during the course of the experiments. Thus, not only the introduction of negative charges by phosphorylation of monomeric Aβ, but also differential aggregation could underlie the distinct association with neuronal membranes, vesicular distribution, and the functional effects of the Aβ phosphorylation-state variants.

Together, the combined data demonstrate the complexity of functional effects caused by intraneuronal accumulation of distinct Aβ variants on autophagy and the endo-lysosomal system. It is interesting to note that disturbance of autophagy and lysosomal function in AD pathogenesis could also involve Aβ independent mechanisms. For example, it has been shown that a loss of PSEN (presenilin) proteins also results in accumulation of autophagic and endo-lysosomal compartments [72–74]. However, distinct mechanisms, including PSEN dependent regulation of lysosomal pH, release of Ca^2+^ from lysosomes that facilitate fusion with late endosomes or autophagosomes, and transcriptional regulation of lysosomal biogenesis have been proposed. Notably, some of these effects might not be related to the role of PSEN proteins as the catalytic components of the gamma-secretase complex [74–76]. Thus, several Aβ-dependent and -independent mechanisms could contribute to the impairment of endo-lysosomal and autophagy related systems, and it will be important to further dissect their relative contribution to the pathogenesis of AD.

## Materials and methods

### Reagents and antibodies

All chemicals used were purchased from Sigma-Aldrich, Merck, or Carl Roth, and used without purification unless otherwise indicated. List of antibodies used in western immunoblotting, immunocytochemistry and histochemistry are indicated in the Table 1.

**Table 1.** List of antibodies and reagents used in the experiments.

Protein of interest	Antibody Clone/specificity	Provider (Cat. No.)	WB dilution	IHC/ICC dilution
Amyloid β (Aβ)	82E1	IBL International (JP10323)	1:500	1:200
	82E1-Biotin	IBL International (JP10326)		ELISA: 1:1000
Aβ	4G8	Biolegend (800709)	1:500	1:250
	4G8-Biotin	Biolegend (800704)		ELISA: 1:1000
APP and Aβ	6E10	Biolegend (SIG-39340)		1:250
APP (C-terminal specific)	140	In-house [77]	1:500	
nmAβ	7H3D6	In-house [78]		1:100
p-Ser8Aβ	1E4E11	In-house [78]	1:250	1:100
p-Ser26Aβ	5H11C10	In-house [38]	1:250	1:100
	SA6192	In-house [35]	1:250	1:100
EEA1 (early endosome antigen 1)	Anti-EEA1	MBL (PM062) Abcam (ab206860)	1:1000	1:200
RAB7 (late endosomal protein)	Anti-RAB7	Abcam (ab50533)		
LC3B (autophagy protein)	Anti-LC3	MBL International (PM036)	1:1000	1:200
	Anti-LC3B	Cell Signaling Technology (2775)		1:200
SQSTM1/p62	Anti-SQSTM1/p62	Sigma Aldrich (P0067)	1:1000	1:200
LAMP2 (lysosomal protein)	H4B4 (hu)	Iowa Hybridoma bank (H4B4)	1:1000	1:200
	ABL-93c (ms)	Iowa Hybridoma bank (ABL-93c)	1:1000	1:200
	Anti-LAMP2A	Abcam (ab25068)	1:1000	1:200
CTSD (lysosomal aspartic endopeptidase)	Anti-CTSD	Abcam (EPR3057Y, ab75852)	1:1000	
Proton ATPase	Anti-ATPase	Synaptic Systems (109 002)	1:500	
TFEB (transcription factor EB)	Anti-TFEB	Abcam (ab264421)	1:1000	
RAB5 (early endosomal protein)	Anti-RAB5	BD Biosciences (610724)	1:1000	
RAB11 (recycling endosomal protein)	Anti-RAB11	Cell Signaling Technology (3539)	1:1000	
ATG14 (autophagy related 14)	Anti-ATG14	MBL International (PD-026)	1:1000	
ULK1/atg1 (unc-51 like autophagy activating kinase 1)	Anti-ULK1/Atg1	Sigma Aldrich (A7481)	1:1000	
Free ubiquitin	Anti-ubiquitin	Sigma Aldrich (U5379)	1:1000	
ATG5 (autophagy related 5)	Anti-ATG5	Abcam (ab108327)	1:1000	
ATG12 (autophagy related 12)	Anti-ATG12	Cell Signaling Technology (2010)	1:1000	
ATG3 (autophagy related 3)	Anti-ATG3	Cell Signaling Technology (3415S)	1:1000	
ACTB/β-actin	Anti-ACTB/β-actin	Cell Signaling Technology (4967)	1:5000	
GAPDH	Anti-GAPDH	SantaCruz Biotechnology (sc -32,233)	1:5000	
MAP2 (microtubule associated protein 2)	Anti-MAP2	Synaptic Systems (SY-SY) (188004)		1:500
Nuclei/DNA	4,6-diamidino-2-phenylindole (DAPI)	ThermoFisher Scientific (D1306)		1:1000
Alexa Fluor 405/488/546/647 dye-conjugated secondary antibody	Donkey/goat – mouse/rabbit IgG	ThermoFisher Scientific (A-11001, A-21202, A-11035, A-11003, A10040, A-31571, A-31573, A-21245, A-11081, A-11006)		1:2500
Streptavidin-HRP conjugate		Biolegend (1474)		ELISA: 1:5000
Phalloidin-Alexa Fluor 555		ThermoFisher Scientific (A34055)		1:10000

Note: WB: western blotting; ICC: immunocytochemistry; IHC: immunohistochemistry; ELISA: enzyme-linked immunosorbent assay.

### Mice

APP-PSEN1dE9×THY1-YFP and WTxTHY1-YFP (wild type) mice were maintained and handled according to the Declaration of Helsinki and approved by the local ethical committees (LANUV NRW 84–02.04.2017.A226). Mice details are indicated in Table **S1**. Whole-brain homogenates from mice hemibrains were prepared as described previously [34]. Briefly, brain tissue was homogenized with a douncer followed by sonication in sucrose buffer (30% sucrose in phosphate-buffered saline [PBS; 137 mM NaCl, 2.7 mM KCl, 10 mM Na_2_HPO_4_, 1.8 mM KH_2_PO_4_, pH 7.4.]) containing Complete® protease and PhosSTOP® phosphatase inhibitors (Roche, 4906837001 and 04693124001) to isolate soluble protein fraction. Homogenates were cleared by centrifugation at 14,000 *g* for 30 min at 4°C. After centrifugation, the resulting supernatant containing respective protein fractions were aliquoted, saved at − 80°C for further usage and the pellet was re-homogenized in 2% SDS in water (pH 7.3) supplemented with protease and phosphatase inhibitors followed by ultrasonication and centrifuged as described above. The resultant supernatant (SDS soluble fractions) was aliquoted and stored at − 80°C. The SDS insoluble pellet was further resolved in 70% formic acid at 4°C overnight with constant agitation. After centrifugation at 14,000 *g* for 30 min at 4°C, the supernatants were flash frozen and stored at − 80°C. For further analyses via WB or ELISA, FA fractions were neutralized with 1 mM NaOH solution. Samples were boiled in Laemmli sample buffer and used for WB analysis.

### Immunohistochemistry

Immunofluorescence staining of mouse brains was performed on 30μm coronal brain sections fixed in 4% paraformaldehyde (PFA) in PBS as described previously [35,79]. Brain tissue sections mounted on glass slides, were washed twice with PBS, and then subjected to antigen retrieval methods using reveal decloaker (Biocare Medical, RV1000M) followed by permeabilization with 0.25% Triton X-100 (Carl Roth, 3051–2) in PBS for 10 min. Sections were stained with X-34 (Sigma Aldrich, SML1954; 0.5 µg/mL) in 60% isopropanol (ISP) in PBS for 10 min. Sections were then washed thrice with 60% ISP-PBS solution. Non-specific binding sites were blocked by treatment with 5% normal horse serum (Life Technologies, 16050122-NZ) and 2.5% bovine serum albumin (BSA; Carl Roth, 8076–3) in PBS, before addition of the primary antibodies. Mouse on Mouse (M.O.M) blocking reagent (Vector Laboratories, MKB-2213-1) was used for primary antibodies generated in mice or rats (dilutions: 1 drop/10 mL). Sections were incubated overnight in a humid chamber at 4°C with the respective dilutions of primary antibodies followed by subsequent washing steps with PBS thrice and blocking buffer twice. Sections were then incubated with an appropriate fluorescently tagged secondary antibody solutions in blocking buffer for 2 h at room temperature (RT). After incubation, tissue sections were washed in PBS thrice, and glass coverslips were mounted using VECTASHIELD Antifade mounting medium with DAPI (Vector laboratories, H-1000-10).

### Preparation of Aβ peptides

Synthetic non-phosphorylated Aβ (npAβ), and phosphorylated Aβ (p-Ser8Aβ and p-Ser26Aβ) peptides (1 mg) were purchased from Peptide Specialty Laboratories GmbH as lyophilized powders and were stored at −20°C. Lyophilized peptides were dissolved in 10 mM NaOH buffer to a stock concentration of ~230 μM, sonicated for 10 min, flash frozen and stored in −20°C until further use.

### Cell culture

Mouse primary cortical neurons were obtained from wild-type C57BL/6 mouse pups (E13-E18). Brain tissue was dissected, and the cortical region was dissociated in trypsin (Life Technologies, 25300104). Post-trypsinization, cells were seeded in Basal Medium Eagle (BME; Life Technologies, 41010026) supplemented with 1× B-27 nutrient supplement (Life Technologies, A1486701), 1% fetal calf serum (FCS; PAN-Biotech, P30–3306), 2 mM L-glutamine (Life Technologies, 25030081). Next day, media was discarded to remove unattached cells and fresh media was added, cells were then maintained and cultured in neurobasal (Life Technologies, 21103049) supplemented with 1% FCS, 1% penicillin-streptomycin solution (PS; 50 U/ml penicillin, 50 µg/ml Streptomycin; Life Technologies, 15140122), 2 mM L-glutamine, at 37°C in a 5% CO_2_ humidified atmosphere until DIV14. Human neuroblastoma SH-SY5Y cells (ATCC, CTR-2266) were cultured in DMEM/F-12 (Life Technologies, 10565018) supplemented with 10% FCS, 1% PS, 2 mM L-glutamine, 1% sodium pyruvate (Life Technologies, 11360039) and 1% non-essential amino acids (Life Technologies, 11140050). Cells were maintained at 37°C in a 5% CO_2_ humidified atmosphere with a media change every alternate day. SH-SY5Y cells were used within passage number 8–13 and were split with 0.5% trypsin-EDTA in a 1:10 ratio.

### Transfection of SH-SY5Y cells with reporter constructs

SH-SY5Y cells were transfected with either GFP-LC3-LC3Δ-RFP or mCherry-GFP-LC3B tandem constructs (0.5 μg/well +1 µl transfection reagent − 24 well plate) with Lipofectamine 2000 (Life Technologies, 11668019) in OptiMEM (Life Technologies, 11058021) in DMEM/F-12, FCS^−^ PS^−^ media. After 8 h, FCS^−^ PS^−^ media was changed to normal culture media, respectively. Transfection efficiency was around 50–60% in all analyzed coverslips.

### Cell treatment with Aβ

Primary cortical neurons (DIV14) were treated with 500 nM of monomeric/unaggregated Aβ variants (npAβ, p-Ser8Aβ and p-Ser26Aβ) in neurobasal, FCS^−^ PS^−^ media. SH-SY5Y cells seeded on D1, were treated with Aβ variants (1 μM) on D3 in DMEM/F-12 media without serum and antibiotics. Control treatment included treatment with equal volume of 10 mM NaOH solution used for preparing the Aβ stock solutions, in respective cell culture medium. SH-SY5Y cells, after 16 h of transient expression of the reporter constructs, were treated with different Aβ variants, autophagy modulator substrates and respective controls in DMEM/F-12, FCS^−^ PS^−^ media for the mentioned time points. After treatment, cells were washed twice with PBS and with 0.025% trypsin-EDTA in PBS to get rid of cell bound Aβ, followed by subsequent washings with PBS again. Cells were either collected by scraping using a cell-scraper or fixed with 4% PFA-PBS for 15 min at RT, for further immunocytochemistry experiments. Collected treatment media was centrifuged at 1000 *g* for 5 min, pellet discarded and supernatant frozen at −20°C until further usage.

### Subcellular fractionation (Figure 3F)

Cells were collected by scraping and mechanically homogenized in hypotonic D buffer (10 mM Tris-HCl, pH 7.4, 10 mM NaCl, 0.1 mM EGTA, 25 mM glycerol 2-phosphate, 1 mM DTT) containing protease and phosphatase inhibitors by repeated suspension through a 23 G × 1” needle. Post-nuclear supernatant (PNS) was collected by centrifugation of the mechanically homogenized cellular material at 2,000 *g* for 5 min. The pellet was the *nuclear material*. Nuclear pellet was then resuspended in Buffer C (5 mM HEPES, 1.5 mM MgCl_2_, 0.2 mM EDTA, 0.5 mM DTT, 0.05% NP40 [Sigma Aldrich, 18876], 30% glycerol, pH 7.9) containing protease and phosphatase inhibitors, ultrasonicated at minimum voltage, three currents in four cycles, followed by repetitive cooling of samples on ice. The *nuclear fraction* was the supernatant collected after centrifugation at 16,100 *g* for 15 min. The PNS collected from the first step was centrifuged at 16,100 *g* for 60 min, and supernatant was used as the crude *cytosolic fraction*. The pellet contained the membrane materials, which were re-homogenized in hypotonic D buffer and again centrifuged at 16,100 *g* for 60 min. The supernatant was discarded, pellet was resuspended in STEN-lysis buffer (20 mM Tris-HCl, pH 7.5, 150 mM NaCl, 1 mM EDTA, 1 mM EGTA, 1% Triton X-100, 2.5 mM sodium pyrophosphate [Sigma Aldrich, P1835], 1 mM β-glycerophosphate [Sigma Aldrich, G9422]) containing protease and phosphatase inhibitors, and the *membrane fraction* was the supernatant collected after centrifugation at 16,100 *g* for 15 min. The crude cytosolic fraction was then further fractionated at 100,000 *g* for 70 min at 4°C under vacuum. The resultant supernatant was the *soluble cytosolic fraction*. The pellet was resuspended in STEN lysis buffer and centrifuged at 16,100 *g* for 15 min. The resultant supernanant represented the *insoluble cytosolic fraction*.

### Enrichment of lysosomes (Figure 5A)

Cells after respective washing steps were collected by scraping and homogenized in isotonic buffer (10 mM Tris-HCl, pH 7.4, 1 mM MgCl_2_, 0.1 mM EGTA, 0.25 M sucrose) containing protease and phosphatase inhibitors, by repeated suspension through a 23 G × 1” needle. Cell homogenate was centrifuged at 2,000 *g* for 5 min. Pellet (nuclear material) was discarded. The post-nuclear supernatant was centrifuged at 16,100 *g* for 15 min. Pellet comprised of lysosome enriched material and the supernatant was used as the crude lysosome depleted material. Pellet was resuspended in STEN-lysis buffer and the *lysosome enriched fraction* was the supernatant collected after centrifugation at 16,100 *g* for 15 min. Supernatant was further centrifuged at 16,100 *g* for 60 min. The resultant pellet contained lysosome depleted material and supernatant was crude *cytosolic material*. The pellet was re-homogenized in STEN-lysis buffer and the supernatant was the *lysosome depleted fraction* collected post-centrifugation at 16,100 *g* for 15 min.

### Immunoprecipitation

Cells after respective washing steps were collected by scraping and homogenized in either isotonic buffer or hypotonic D buffer containing protease and phosphatase inhibitors by repeated suspension through a 23 G × 1” needle. Cell homogenate was centrifuged at 2,000 *g* for 5 min and the nuclear material (pellet) was discarded. The post-nuclear supernatant was then used for IP experiments. Sepharose G beads (ThermoFisher Scientific, 101242) were incubated with PNS material at RT for 1 h, beads were further pelleted down and boiled in 1% SDS in 50 mM Tris-HCl, pH 7.6 along with sample buffer, used as *bead control*. Supernatant exposed to beads only were devoid of nonspecific binding substrates and was used further as *loading control*. New set of beads were precoated with respective primary antibodies for 1 h and then incubated with the PNS solutions overnight at 4°C. Beads were pelleted down and boiled with 1% SDS in 50 mM Tris-HCl along with sample buffer as the *IP eluate*.

### Western immunoblotting

For different cellular fractions prepared as described earlier, the protein content was estimated using the standard Pierce™ BCA protein assay kit (ThermoFisher Scientific, 23225). For direct comparison between the samples, equal amount of protein was loaded. Samples were dissolved in Laemmli buffer and were boiled at 95°C for 5 min. They were loaded on a precast 4–12% NuPage® Bis-Tris gels (Life technologies, NP033–5/6, WG140–2/3). Gels and prestained protein molecular weight markers were purchased from Life technologies. The separated proteins were electro-transferred onto a 0.2-μm nitrocellulose (NT) membrane (Amersham, 1060001) for 1 h 45 min. After blotting, the NT membranes were boiled in PBS for 10 min. Ponceau staining was done to monitor protein loading of samples. The blots were then incubated in 5% skim milk in TBS-T (TBS [50 mM Tris-HCl, pH 7.5, 150 mM NaCl] containing 0.1% Tween 20 [Sigma Aldrich, P1379]) for 1 h at RT. Blots were incubated overnight at 4°C in the primary antibody solution (respective dilutions in TBS-T, Table 1). Blots were washed and incubated with appropriate secondary antibodies conjugated either with HRP or fluorescent dyes for 1 h. Blots were developed either by enhanced chemiluminescence (ECL) or fluorescence imaging. For ECL imaging, blots were incubated with equivalent mixtures of ECL solution A (0.1 M Tris, 0.4 mM coumaric acid, 2.5 mM luminol, pH 8.5) and B (0.1 M Tris, 0.018% H_2_O_2_) mixed prior to application on blots. Signals were detected with an ECL imager (Bio-Rad, Germany). For fluorescence detection, blots were imaged on a Li-COR imaging station (Li-COR Biosciences, Germany). Blots were presented as is or with minimal linear contrast enhancement (Figure 5B–D; **S1**; **S3E**; **S5E, S8E**). Quantification of band signals was done using ImageJ – Gel processing module or Image Studio processing software (LiCOR Biosciences, Germany). All samples were analyzed in biological duplicates in two or three independent experiments as indicated in the respective figure legends. Values from each sample were normalized to the values from the control cells in that experiment and expressed as fold-change as indicated in the respective graphs (Figure 7E–F; **8B-F**; **S5A-D**, **F, G**) and Table **S3**.

### Elisa

Plates (ThermoFisher Scientific, 44-2404-21) were coated with either anti-Aβ (82E1, 4G8), anti-npAβ (7H3D6) or anti-pAβ antibodies (p-Ser8Aβ − 1E4E11 and p-Ser26Aβ − 5H11C10 (0.1 μg/well) as capture antibody at RT for 2 h and blocked with 1 mg/ml BSA solution in PBS. Equal protein amounts of different mouse brain fractionated material or cellular fractions were then added to the coated plates as antigen solutions and incubated at 4°C for 16 h. After incubation, residual liquid from the plate was removed by gently tapping the plates. 200 µl of blocking buffer (1 mg/ml BSA) was added per well and incubated at 25°C for 2 h. Wells were further tapped dry and incubated with 4G8-biotin/82E1-biotin detection antibodies (0.05–0.1 μg/well) at RT for 2 h followed by subsequent washing and incubation with streptavidin conjugated HRP complex (1:5000) for another 2 h. Wells were then washed thoroughly four times and filled with 100 µl of 3,3,5,5-tetramethylbenzidine substrate (TMB; ThermoFisher Scientific, N301) in each well and incubated at 25°C until sufficient blue color developed (time ranged from 2–15 min depending on the different capture antibodies used in the experiment). 100 µl of stop solution (4 M H_2_SO_4_) was added to each well. Absorbance values from each well were read at a Tecan plate reader at a wavelength of 450 nm and background measurement at 620 nm. Multiple readings were recorded for a single well and averaged. In each experiment, each sample was analyzed in technical duplicate/triplicate wells as indicated and average values from an independent experiment are presented (Figures 1E, 3G, **SA-D** and Table **S2**).

### Immunocytochemistry

Cells were cultured on glass coverslips and treated similarly as mentioned above. After treatment, cells were subsequently washed and fixed with 4% paraformaldehyde in PBS for 15 min. Cells were then permeabilized with 0.25% Triton X-100 in PBS for 1 min, followed by blocking with 2.5% BSA, 0.125% Triton X-100 in PBS. Incubation with primary antibodies (respective dilutions in blocking buffer) was performed overnight at 4°C. Next day, coverslips were washed and incubated with respective secondary antibodies (respective dilutions in blocking buffer) for 1 h at RT. Removal of the secondary antibody solution was followed by washes with PBS (thrice) and distilled water (once) and coverslips were mounted on the slides using ImmuMount (ThermoFisher Scientific – Shandon Epredia™, 9990412). For surface staining without permeabilization, Triton X-100 was not used in any solution during the staining procedure.

### Microscopy

Confocal images depicted in figure 1A; Figure 2A–E; Figure 4A–E; **S2A**, **C**, **E-I**; **S4A**, **C**; were acquired on VisiScope CSU-W1 spinning disk confocal microscope using VisiView Software (Visitron Systems GmbH, Germany). Laser power, detector gain and other parameter settings were kept constant for the acquisition of each set. Each immunostaining was performed with cross-combination of secondary antibodies to check nonspecific reactivity. Images were acquired using either 5×, 20×, 40× water or 63× water immersion objective, 2048 × 2048 pixels, 1 × 1 binning. *z*-stacks were obtained at 20×, 40× or 63× magnification, 2,048 × 2,048 resolution, steps = 16, and step size = 1 μm. Imaging was also done with a Carl Zeiss Axio Imager 2 ApoTome fluorescence microscope (Zeiss, Germany) using a 63× oil immersion objective (Figure 3A; **6A**, **C**; **7A**; **S6C**, **E**) or BZ-X800 fluorescence microscope (Keyence, Germany) using a 20× or 40× air objective (quantification for Figure 3B–E). Per coverslip, randomly selected 6–10 images were captured, which were further used for quantification – representative of ~150–200 cells per treatment condition or colocalization analysis. All images depicted herewith were minimally processed using Fiji ImageJ software.

### Electron microscopy

SH-SY5Y cells were seeded and treated on glass coverslips. After treatment cells were washed and fixed with 4% EM grade PFA (methanol free) and 2.5% glutaraldehyde in ddH_2_O overnight at 4°C. For images depicted in figure **S6C, E**, procedure mentioned in the immunocytochemistry section was followed. For EM, fixed cells were washed in 0.1 M cacodylate buffer (pH 7.4). Samples were incubated in 1% osmium tetroxide, 0.8% potassium ferricyanide in 0.1 M cacodylate buffer (pH 7.4) for 2 h at RT, followed by washing for 10 min in 0.1 M cacodylate buffer (pH 7.4) thrice. Osmified cells were then dehydrated in a series of increasing ethanol solutions (30, 50, 70% for 10 min each, 0.5% uranyl acetate in 70% ethanol for 60 min, 90, 95, and 100% ethanol 10 min each) at RT. Further, cells were infiltrated with a 1:1 ratio of ethanol and propylene oxide for 10 min at RT and finally 10 min incubation in 100% propylene oxide twice. Cells were then infiltrated with Epon epoxy resin (Sigma-Aldrich, 45359-1EA-F) in increasing ratios of propylene oxide: Epon (1:1 for 1 h, 1:2 overnight at RT), and finally twice with 100% Epon for 1 h at RT, before embedding using beem capsules (Plano, G360–1). Epon was polymerized by curing at 60°C for 48 h. After polymerization, beem capsules were trimmed away with a razor blade and the coverslips were removed using freeze-thaw cycles of liquid nitrogen and a heating plate set to 60°C. Once the coverslips were removed, the block face was trimmed to fit on an EM grid (formvar and copper-coated copper slot grid, Science Services Cat. EFCF2010-Cu-50) and ultra-thin sections 50-nm-thick were collected. Sections were counterstained with 1% aqueous uranyl acetate for 25 min and lead citrate Ultrastain solution (Leica, Cat. 16707235) for 7 min with thorough washing and drying in between. Sections were imaged with a Zeiss Crossbeam 550 (acceleration voltage: 30 keV, probe current: 150 pA) using a STEM detector. For all examined samples, multiple cells were imaged (>4) with resolution between 24 nm and 2.4 nm per pixel, across each EM grid. This ensured classification of different cellular morphology and avoided inadvertent production of a biased/subjective data selection. Best representative images (*N* = 3) have been illustrated in figure **S6A**, processed using Fiji ImageJ software. Quantification of the number of autophagic compartments (Figure **S6B**) was done manually using a 10 µm^2^ ROI grids, placed in the cytosolic region of the individual cells using the “rule of L” counting method. Results are representative of values computed from three independent experiment (n ~30 cells).

### Lysosomal acidification assay using LysoSensor

Cells were cultured on coverslips and treated with Aβ variants (primary cortical neurons, DIV14–500 nM, 4 h; SH-SY5Y cells, D3–1 μM, 24 h) in presence of 50 µM LysoSensor-dextran probe (ThermoFisher Scientific, L7545) in cell culture media for the indicated time points. Cells were washed and stained as described before. For fluorescence analyses, cells were cultured and treated in a 96-well plate for the indicated time points, and washed thrice with PBS to remove the excess probe. Fluorescence intensity analysis of blue and green channel was done using a multiplate reader (Tecan Life Sciences, Switzerland). of the relative fluorescence emission intensity was computed from the fluorescence values measured at λ_Ex_ 340 nm and λ_Em_ 450 nm *(blue channel)* and λ_Em_ 535 nm *(green channel)*, respectively. Values were normalized to absorbance at 340 nm and protein content per well, and represented as change of LysoSensor acidification with respect to control cells (Figure 7B and **S7C-D**). pH values were computed by preparing a standard curve, wherein cells treated with 1 µM nigericin (Sigma Aldrich, N7143) and 1 µM monensin (Sigma Aldrich, M5273) were incubated with media of pH 4.5, 7.5 and 9.2. As independent controls, 100 nM bafilomycin A_1_ (Sigma Aldrich, 553210), 1 mM NH_4_Cl- and 10 µM rapamycin (Cayman Chemicals, 19438)-treated cells were also used as independent controls to validate the measurements. Change in LysoSensor pH (ΔpH) for Aβ treated cells was quantified using the formula ΔpH = pH_final_ (control/Aβ, *t* = 24 h) − pH_initial_ (control, *t* = 0 h). Box plots (Figure 7C and **S7E**) depict the overall distribution of data, and each data point represents average values from an independent experiment; *n* = 12, *N* = 4.

### CTSD and CTSE assay

Cells were seeded in a 96-well plate (12000 cells/well, D1) and treated with different Aβ variants (primary cortical neurons, DIV14–500 nM, 4 h; SH-SY5Y cells, D3–1 μM, 24 h) in neurobasal, FCS-/PS- media. After the treatment, the supernatant was discarded, and cells were washed with PBS once. Cells were lysed by solubilizing the plasma membrane and intracellular membranes by adding 50 μL of 200 μg/mL digitonin in acetate buffer to the wells followed by incubation on ice for 15 min. Additionally, 50 μL of the Omnicathepsin D & E fluorogenic substrate (Enzo Life sciences, BML-P145) in sodium acetate buffer was added to the lysates in the wells for a final concentration of 30 μM of substrate and 10 mM DTT. Plate was incubated for 25 min at RT and the reaction was stopped by addition of 10 µl of ethanol. The relative amount of substrate cleavage in each well was computed using multiplate reader at λ_Ex_ 380 nm and λ_Em_ 460 nm. Values were normalized and represented as fold-change with respect to control cells. Graphs in Figure 7G and S7F represent data from 3 independent experiments (*n* = 9).

### Image analyses

All images were processed and analyzed using Fiji ImageJ software. Mander’s overlap coefficient (Figure 1B–D; Figure 2B–F; Figure 4B–F; Figure 6E; **S2B**, **D**; **S4B**, **D**; **S6D**, **F**) analysis was done using the colocalization processing module of the Fiji ImageJ plugin. Distance from the plaque core (Figure 1B) was measured using the concentric circles plugin module. Fluorescence intensity of the green channel (Figure **S3A-B**), and the number of neurons positive for Aβ signals (Figure **S3-D**) were quantified using the processing module of Keyence software. Intensity analysis (Figure **S4E-I**) was performed by manually selecting the region of interest (ROI) and measuring the intensity of the individual channels in the selected area. RFP/GFP (Figure 6A) and LysoSensor Green/Blue (Figure 7A) ratiometric images were created by using the image calculator module. Intensity of green and red channel for a particular ROI was calculated and ratios of the individual values were computed (Figure 6B and **S7A**). For computing the number of mCherry^+^GFP^±^ vesicles or per cell (Figure 6D and **S7B**), cell outline was drawn manually and number of vesicles per cell (nucleus count – DAPI) was calculated. Similarly, the number of perinuclear and peripheral LAMP2-positive lysosomes or percentage of LAMP2 compartments containing Aβ was computed using the concentric circle processing module (Figure 7D). Due to slight variability in transfection efficiency or the number of cells in different experimental set-ups, the readings from each experiment were averaged and normalized to the control cells from that particular set. Average values from each experiment were normalized to the respective controls in the same set. Values from independent experiments were then computed and values are represented as independent data points.

### Statistical analyses

All graphical illustrations have been prepared using GraphPad Prism 9.5.1.733. Statistical analysis for a particular graph is mentioned in the respective figure legends. All tests have been performed using the in-built default settings of the module in the software without modifications unless mentioned otherwise; not significant (ns) *p* > 0.05; *p = 0.05; **p = 0.01; ***p = 0.001; ****p = 0.0001.

## Supplementary Material

Supplemental Material

## Data Availability

The data of this study is available from the corresponding author (JW) upon reasonable request.
