# Supplementary material for: Phosphorylation-state dependent intraneuronal sorting of Aβ differentially impairs autophagy and the endo-lysosomal system
Source: Autophagy. 2023 Aug 29;20(1):166–87. doi: 10.1080/15548627.2023.2252300 (PMC10761119; doi:10.1080/15548627.2023.2252300)
Supplement: Supplemental Material [file KAUP_A_2252300_SM0352.docx]

**Supplementary Material**

**Phosphorylation-state dependent intraneuronal sorting of Aβ differentially impairs autophagy and the endo-lysosomal system**

Akshay Kapadia^a^, Sandra Theil^a^, Sabine Opitz^b,c^, Nàdia Villacampa^b^, Hannes Beckert^d^, Susanne Schoch^c^, Michael. T. Heneka^b,e^, Sathish Kumar^a^ and Jochen Walter^a^*

^a^ Department of Neurology, University Hospital Bonn, Bonn, Germany

^b^ Neuroinflammation Unit, German Center for Neurodegenerative Diseases e. V. (DZNE), Bonn, Germany

^c^ Section for Translational Epilepsy Research, Department of Neuropathology, University Hospital Bonn, Bonn, Germany

^d^ Microscopy core facility, University Hospital Bonn, Bonn, Germany

^e^ Department of Neurodegenerative Disease and Geriatric Psychiatry, University Hospital Bonn, Bonn Germany

**Contact** Jochen Walter [Jochen.Walter@ukbonn.de](about:blank) Department of Neurology, University Hospital Bonn, 53127 Bonn, Germany.

**
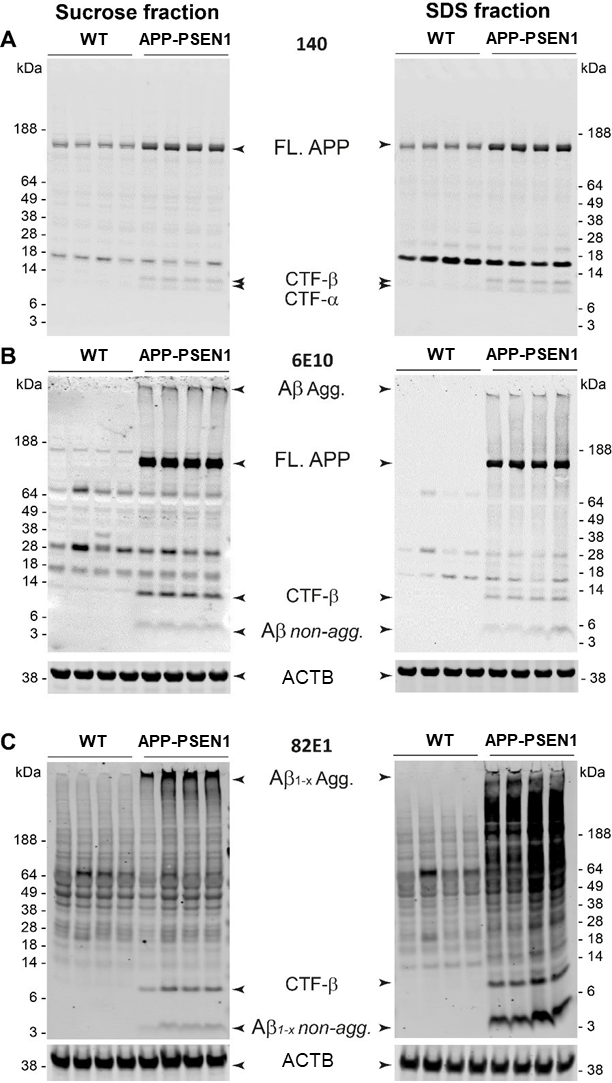
**

**Figure S1.** Expression of human APP and accumulation of Aβ in APP-PSEN1 transgenic mice. (**A-C**) Western immunoblotting analyses of sucrose soluble and SDS soluble fractions of wild type WTxTHY1-YFP and transgenic APP-PSEN1dE9xTHY1-YFP mouse brains (see Table **S1**, mouse ID 1-8). (**A, B**) APP and APP derivatives were detected in parallel with polyclonal antibody 140 recognizing the C terminus of APP and monoclonal antibody 6E10 recognizing an N-terminal epitope of the Aβ domain. Primary antibodies 140 (**A**) and 6E10 (**B**) were detected by anti-rabbit IR-680 conjugated and anti-mouse conjugated IR-800 secondary antibodies, respectively, using LiCOR fluorescence imaging. While antibody 140 recognizes mouse endogenous as well as transgenic human APP, antibody 6E10 selectively detects transgenic human APP. Migration of full-length (FL) APP, APP C-terminal fragments (CTF), aggregated and non-aggregated Aβ is indicated. (**C**) Aβ species and APP β-CTF were detected with anti-Aβ_1-x_ antibody (82E1). This antibody recognizes a neoepitope generated by BACE/β-secretase mediated cleavage of APP, and thus, does not recognize FL APP. ACTB was used as loading control respectively.


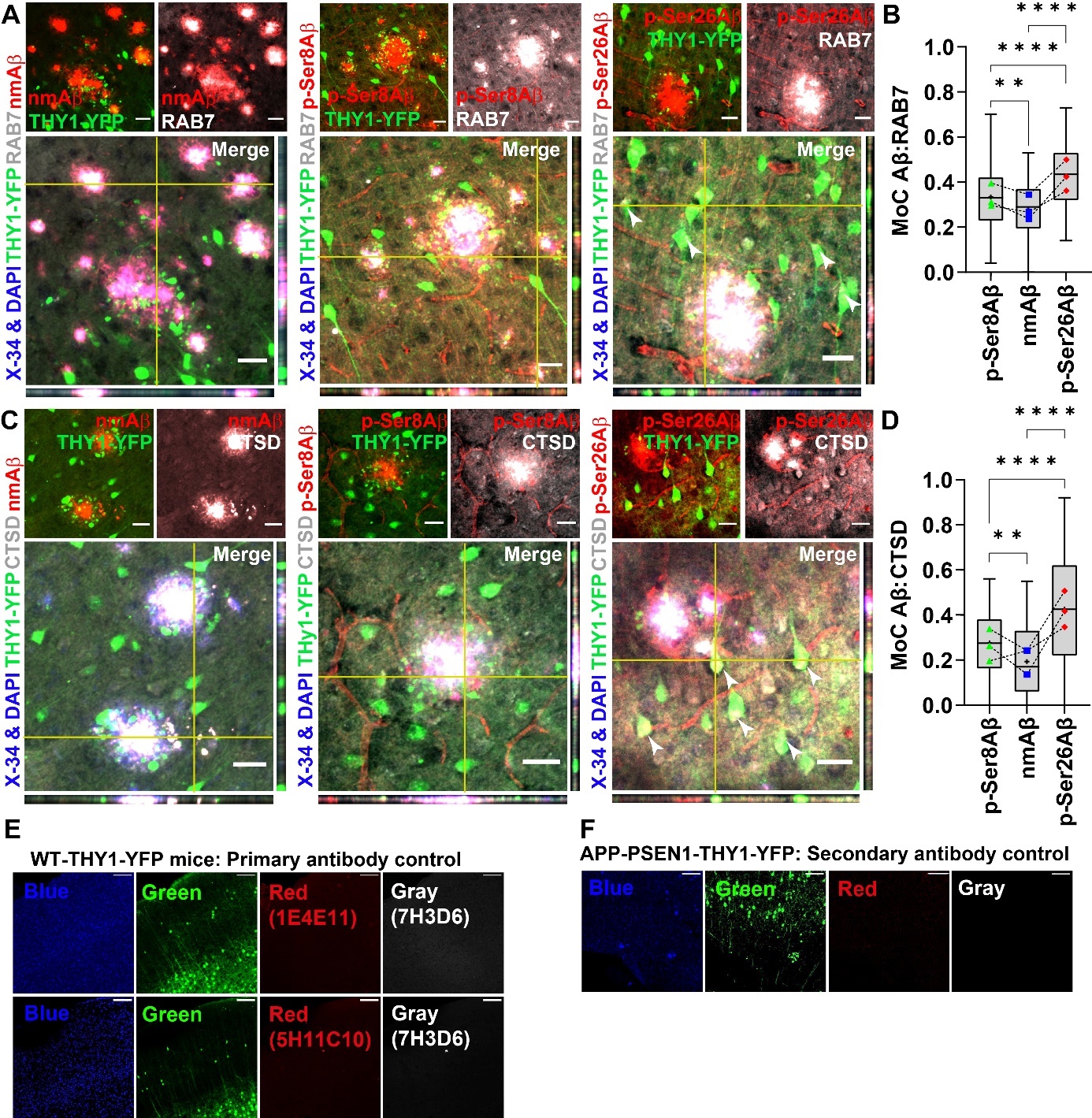


**Figure S2.** Intraneuronal colocalization of pAβ with late endosomal and luminal lysosomal marker proteins in APP-PSEN1 transgenic mice. (**A, C**) Immunohistochemical staining of late endosomal protein RAB7 (**A**, *gray channel*) and lumenal protease CTSD (**C**, *gray*) along with different phosphorylation state specific Aβ antibodies (nmAβ: 7H3D6; p-Ser8Aβ: 1E4E11 and p-Ser26Aβ: 5H11C10, *red channels respectively*) in brain sections of APP-PSEN1dE9xTHY1-YFP transgenic mouse cortex. Blue channel depicts staining of nuclei and fibrillar Aβ with DAPI and X-34, respectively. Scale bar: 50 µm. White arrowheads indicate colocalized punctate staining between red and gray channels in THY1-YFP positive neurons. (**B, D**) Mander’s coefficient of overlap (MoC) between red channels (npAβ, *blue*; p-Ser8Aβ, *green* and p-Ser26Aβ, *red data points*) with respect to gray channels (RAB7, **B** and CTSD, **D**), quantified within THY1-YFP positive neurons respectively. Box plot depicts the overall distribution of data, and each data point represents average values from an individual mouse, N = 3 transgenic mice. Values represent mean ± S.E.M. * *p* = 0.05; ** *p* = 0.01; *** *p* = 0.001; **** *p* = 0.0001 (One-way ANOVA, GraphPad Prism). (**E**) Representative images for primary antibody controls, depicting the cortical regions of WTxTHY1-YFP non-transgenic mouse stained for p-Ser8Aβ (1E4E11, *red*) or p-Ser26Aβ (5H11C10, *red*), along with nmAβ (7H3D6, *gray*) and DAPI + X-34 (nuclei + plaque core, *blue*). Scale bar: 100 µm. **F.** Representative images for secondary antibody controls, depicting the cortical regions of APP-PSEN1dE9xTHY1-YFP transgenic mouse stained with donkey anti-mouse IgG - Alexa Fluor™ 546 (*red*) and donkey anti-rat IgG - Alexa Fluor™ 647 (*gray*) along with DAPI + X-34 (nuclei + plaque core, *blue*). Scale bar: 100 µm.


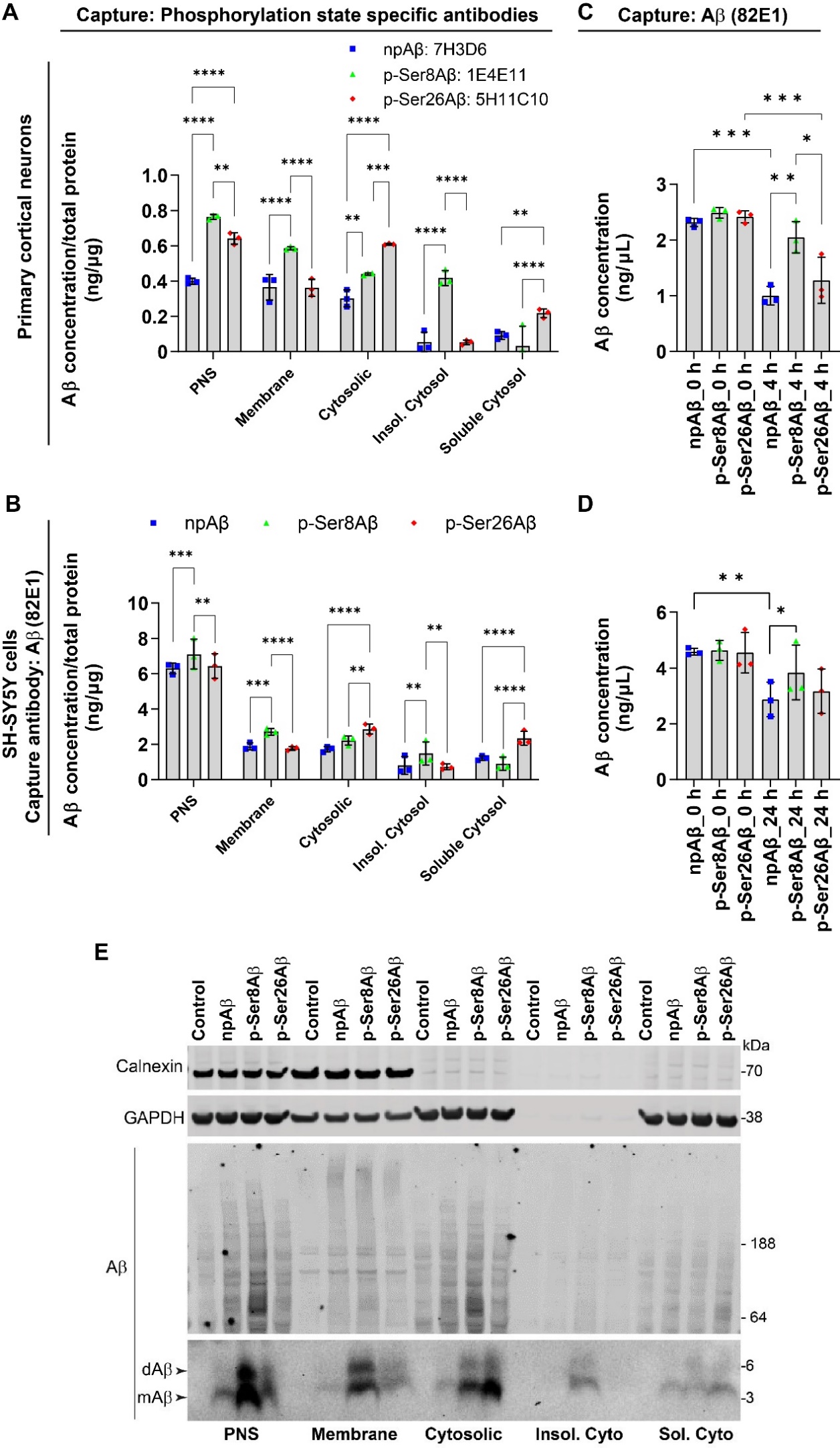


**Figure S3.** Phosphorylation-state dependent intracellular uptake and accumulation of Aβ. (**A-D**) Bar plots depicting the quantification of Aβ concentration by ELISA in different cellular fractions (**A, B**) and in treatment media (**C, D**) of primary cortical neurons (**A, C**) and SH-SY5Y neuroblastoma cells (**B, D**). Aβ species were captured with either phosphorylation state specific antibodies (**A**); npAβ (7H3D6); p-Ser8Aβ (1E4E11); p-Ser26Aβ (5H11C10) or generic Aβ (82E1, **B-D**) antibodies, respectively. Bound Aβ detected with either Aβ detection antibodies (**A**, 82E1-biotin and **B-D**, 4G8-biotin), respectively. Values represent mean ± S.D., n = 9, N = 3. * *p* = 0.05; ** *p* = 0.01; *** *p* = 0.001; **** *p* = 0.0001 (A-B, Two-way ANOVA; C-D, One-way ANOVA, GraphPad Prism). (**E**) NuPAGE and western immunoblot analyses of Aβ in different cellular fractions of primary cortical neurons treated with the indicated Aβ variants (500 nM, 4 h). Aβ was detected with anti-Aβ (82E1) antibody. Contrast in the upper panel was increased lineally with the xy tool better visualize higher molecular weight Aβ assemblies.

**
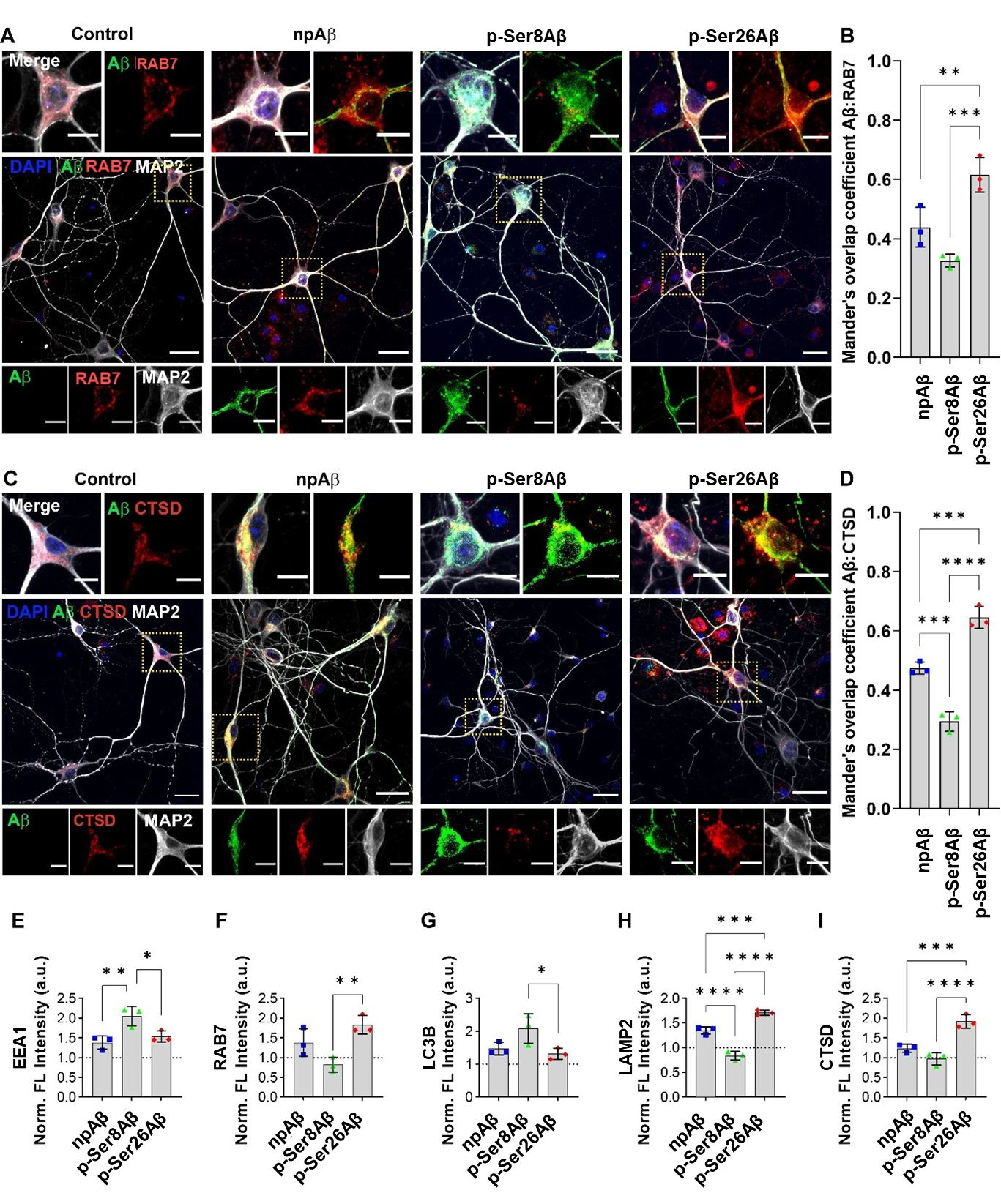
Figure S4.** Phosphorylation-state specific intraneuronal sorting of Aβ to late endosomal and lysosomal compartments. (**A, C**) Primary cortical neurons were incubated with the indicated Aβ variants (500 nM, 4 h) and co-stained with antibodies against the microtubule-associated protein 2 (MAP2, *gray*), Aβ (82E1, *green*) and RAB7 (**A**, *red*) and CTSD (**C**, *red*). Nuclei were additionally stained with DAPI (*blue*). Scale bar: 10 µm. Dotted boxes indicate the region zoomed in the merged panels (*above*) and individual channels (*below*). (**B, D**) Mander’s overlap coefficients for Aβ (*green channel*) and RAB7 (*red channel*, **B**) or Aβ and CTSD (*red channel*, **D**) was determined by the FiJi ImageJ colocalization processing module. Values represent mean ± S.D.; n = 6, N = 3. (**E-I**) Bar plots depicting the densitometric quantification of the normalized values of fluorescence intensity of red channels - EEA1 (**E**); RAB7 (**F**); LC3 (**G**); LAMP2 (**H**) and CTSD (**I**); stained primary cortical neurons treated without (control) or with indicated Aβ variants (500 nM, 4 h). Representative images: Fig. 4A, C, E, and SI Fig. S4A, C; respectively. Fluorescence intensities were normalized to that of control cells (*dotted line*). Values represent mean ± S.D.; ~80 - 100 neurons, n = 6, N = 3. * *p* = 0.05; ** *p* = 0.01; *** *p* = 0.001; **** *p* = 0.0001 (One-way ANOVA, GraphPad Prism).


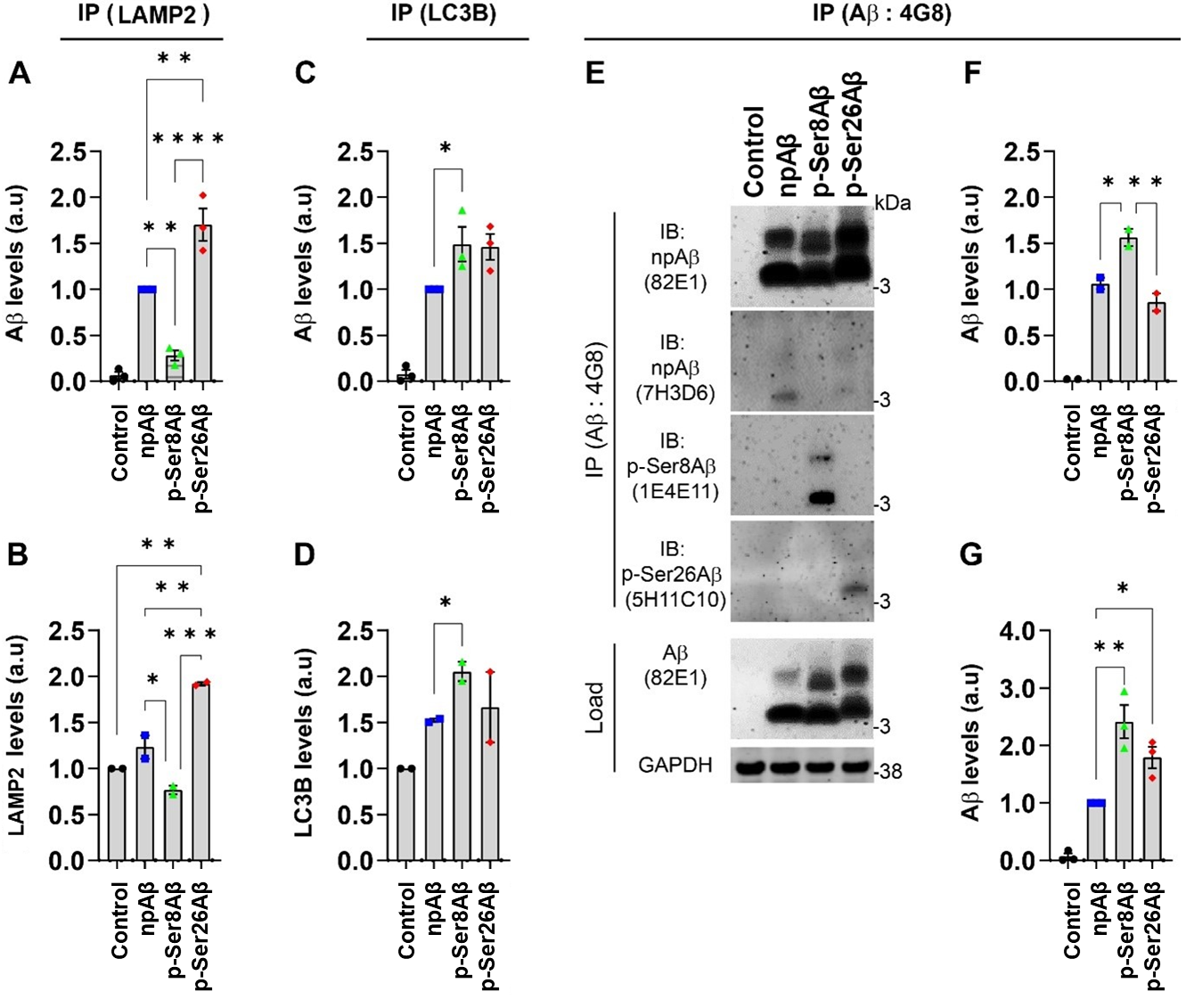
**Figure S5.** Phosphorylation-state dependent vesicular localization and intracellular accumulation of Aβ. (**A-D**) Primary cortical neurons treated with Aβ variants (500 nM, 4 h) were homogenized in isotonic buffer and intact lysosomes or autophagic vesicles were immunoisolated respectively (Fig. 5B-C). Bar plots depicting the relative levels of Aβ in immunoisolated lysosomes (**A**) or autophagic vesicles (**C**) along with the quantification of LAMP2 (**B**) and LC3B (**D**) in the IP eluates respectively. (**E**) Primary cortical neurons treated with Aβ variants (500 nM, 4 h) were homogenized in hypotonic buffer and Aβ from the PNS was immunoprecipitated with anti-Aβ antibody 4G8. Aβ in the individual fractions was then detected using anti-Aβ antibody 82E1 or the indicated phosphorylation-state specific antibodies via western immunoblotting. GAPDH and Aβ (82E1) were used as loading/starting controls. IP, immunoprecipitation; IB, immunoblot. (**F, G**) Bar plots depicting the relative Aβ levels in the IP eluates detected with phosphorylation state specific antibodies or generic Aβ_1-x_ antibody, respectively. Values represent mean ± S.E.M.; n = 9, N = 3. * *p* = 0.05; ** *p* = 0.01; *** *p* = 0.001; **** *p* = 0.0001 (One-way ANOVA, GraphPad Prism). All results and analyses are representative of three independent experiments.

**
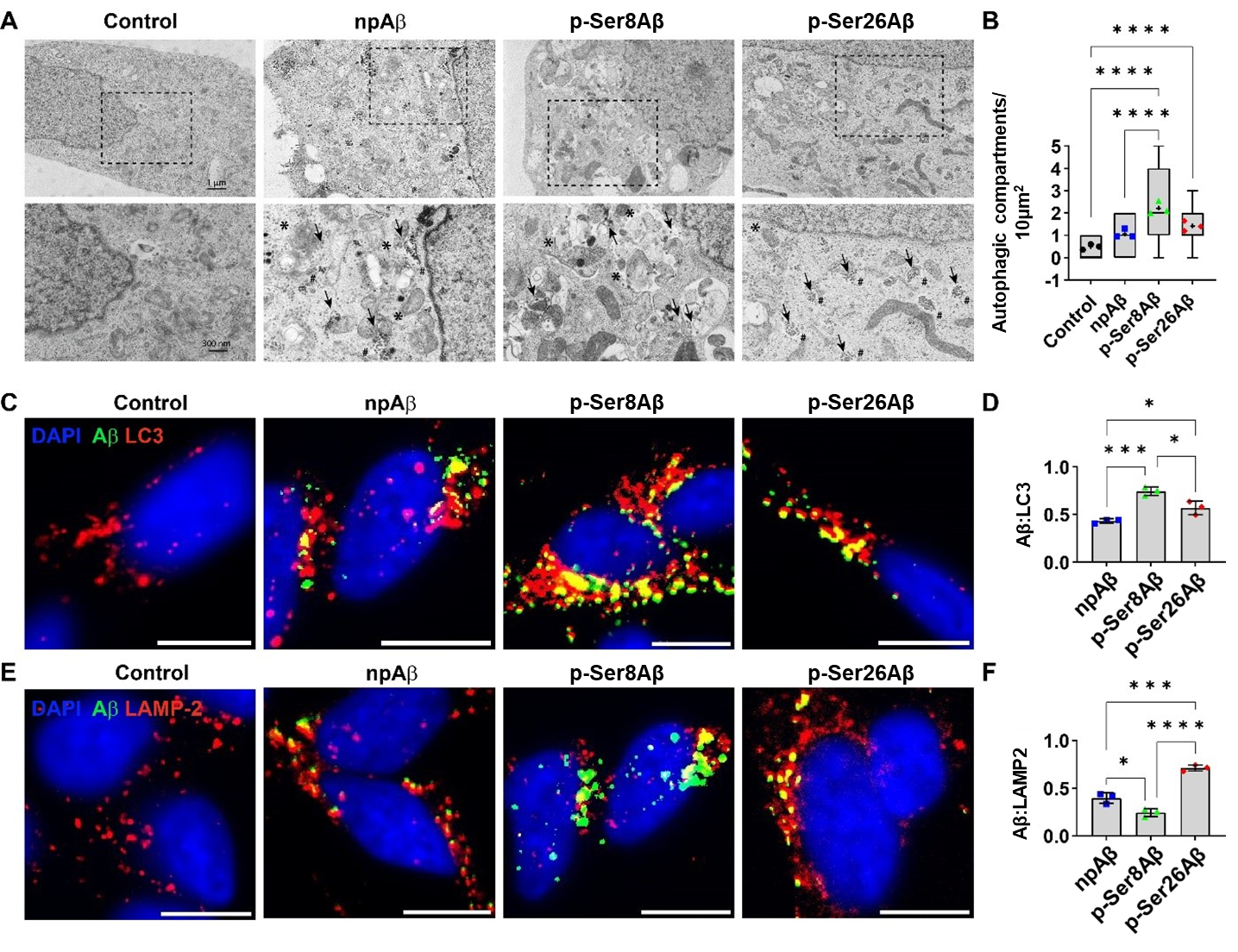
Figure S6.** Phosphorylation-state specific sorting of Aβ to autophagic and lysosomal compartments. (**A**) Scanning transmission electron microscopy (STEM) of SH-SY5Y cells treated without (control) or with Aβ variants (1 μM, 24 h); Scale bar: 1 μm. Dotted rectangles indicate the region zoomed in the panels below (Scale bar: 300 nm). Arrows indicate electron dense structures; asterisks indicate probable autophagosomes; hashtags indicate probable autolysosomes. Images are representative of three independent experiments. (**B**) Box plot depicting quantification of autophagic components in the ROI (10 μm^2^) within the cytoplasm of SH-SY5Y cells treated with Aβ variants (1 μM, 24 h). Box plot depicts the overall distribution of data, and each data point represents average values from independent experiments; n ~ 30 cells, N = 3. (**C, E**) Immunocytochemical staining of LC3 (**C**) and LAMP2 (**E**) in SH-SY5Y cells upon incubation without (control) or with the indicated Aβ variants (1 µM, 24 h). Cells were stained with anti-Aβ antibody 82E1 (*green*), and antibodies against the respective vesicular markers (*red*) along with DAPI (nuclei, *blue*). Scale bar: 5 µm. (**D, F**) Mander’s overlap coefficients of Aβ (*green channel*) and LC3 (*red channel*, **D**) or Aβ and LAMP2 (*red channel*, **F**) was determined by the FiJi ImageJ colocalization processing module. Values represent mean ± S.D.; n = 6, N = 3. * *p* = 0.05; ** *p* = 0.01; *** *p* = 0.001; **** *p* = 0.0001 (One-way ANOVA, GraphPad Prism).

**
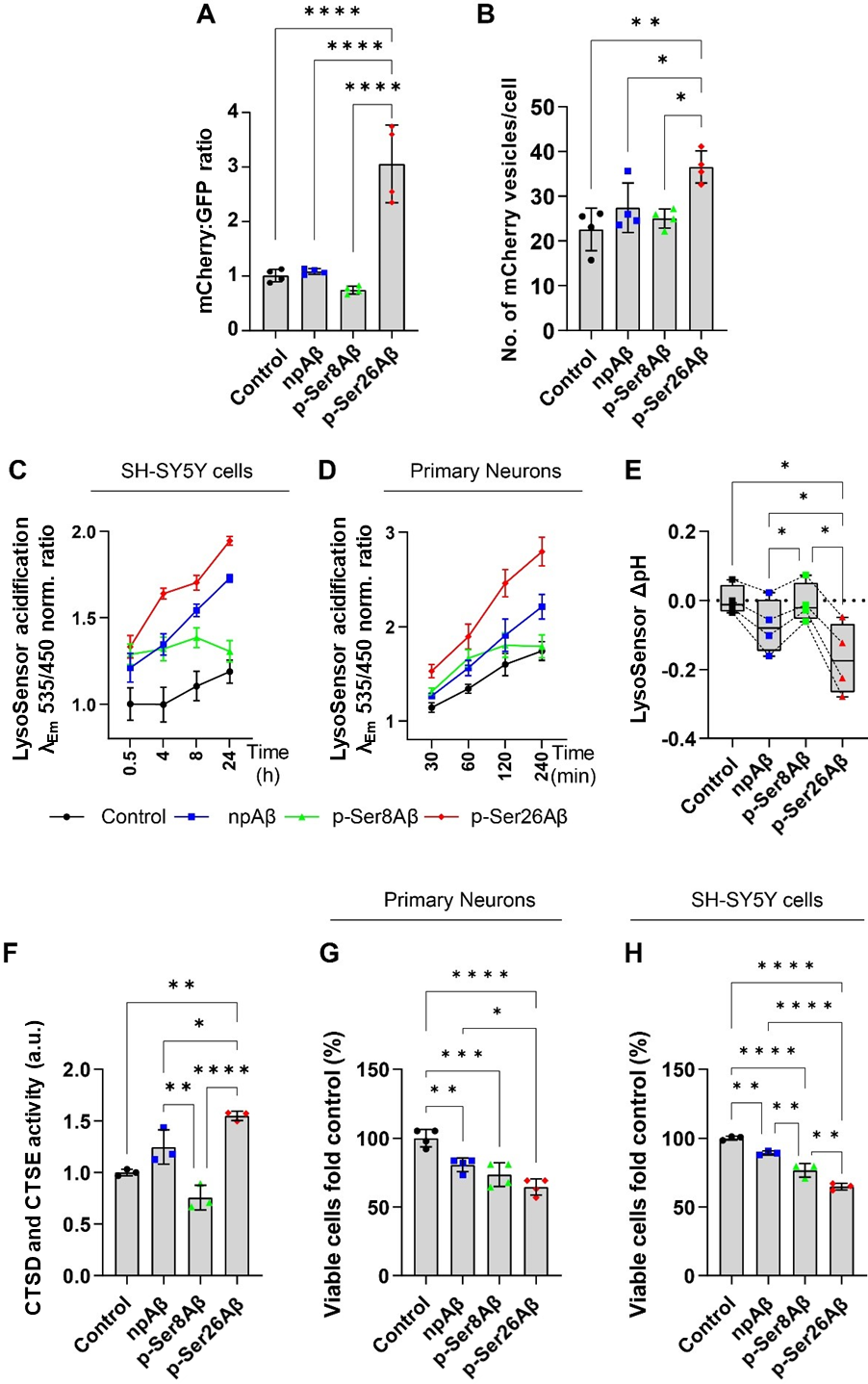
**

**Figure S7.** Phosphorylation-state dependent effect of Aβ on lysosomes and cell viability. (**A, B**) SH-SY5Y cells expressing tandem reporter constructs for autophagic flux (mCherry-GFP-LC3B) upon treatment with different Aβ variants (1 µM, 24 h) were analyzed by immunocytochemistry. **A.** Bar plot showing the ratio of mCherry:GFP fluorescence intensities, respectively. **B.** Quantification of the number of mCherry positive vesicles per cell. Readings were normalized to control cells incubated without Aβ. Values represent mean ± S.D.; n = 8, N = 4. * *p* = 0.05; ** *p* = 0.01; *** *p* = 0.001; **** *p* = 0.0001 (One-way ANOVA, GraphPad Prism). (**C, D**) Line plots representing time-dependent lysosomal acidification. SH-SY5Y cells (50 μM LysoSensor + 1 μM Aβ variants, **C**) and primary cortical neurons (50 μM LysoSensor + 500 nM Aβ variants, **D**) were analyzed in a multi-well plate reader at the mentioned time points. Readings were normalized to cells incubated only with the LysoSensor. Fluorescence emission intensities were measured at λ_Ex_ 380 nm and ratio at λ_Em_ 535, and 450 nm was computed. Values were normalized to control cells and presented as mean ± S.E.M.; n = 9, N = 3. (**E**) Change in LysoSensor pH (ΔpH) for primary cortical neurons (50 μM LysoSensor + 500 nM Aβ variants, 4 h) was quantified using the formula ΔpH = pH_final_ (control/Aβ, t = 24 h) − pH_initial_ (control, t = 0 h). Box plot depicts the overall distribution of data, and each data point represents average values from an independent experiment; n = 12, N = 4. * *p* = 0.05; ** *p* = 0.01; *** *p* = 0.001; **** *p* = 0.0001 (Repeated measures one-way ANOVA, GraphPad Prism). (**F**) Primary cortical neurons treated with different Aβ variants (500 nM, 4 h) were examined for CTSD and CTSE activity (per µg of total cellular protein) using a CTSD and CTSE cleavable fluorogenic substrate. Readings were normalized to control cells; values represent mean ± S.D.; n = 6, N = 3. (**G, H**) Cell viability of primary cortical neurons treated with different Aβ variants (500 nM, 4 h, **G**) or SH-SY5Y neuroblastoma cells (1 µM, 24 h, **H**) was examined using CellTiter-Fluor™ cell viability assay kit. Cells were seeded in 96-well plates (10000-15000 cells/well, D1) and were treated with different Aβ variants at DIV14 (primary cortical neurons) or D3 (SH-SY5Y cells) in neurobasal or DMEM/F12, FCS^-^PS^-^ media. After treatment, procedure was followed as indicated in the manufacturer’s protocol. Plates were read using multiplate reader at λ_Ex_ 390 nm and λ_Em_ 505 nm. Readings were normalized to control cells; values represent mean ± S.D.; primary neurons, n = 8, N = 4; SH-SY5Y cells, n = 6, N = 3. * *p* = 0.05; ** *p* = 0.01; *** *p* = 0.001; **** *p* = 0.0001 (One-way ANOVA, GraphPad Prism).

**
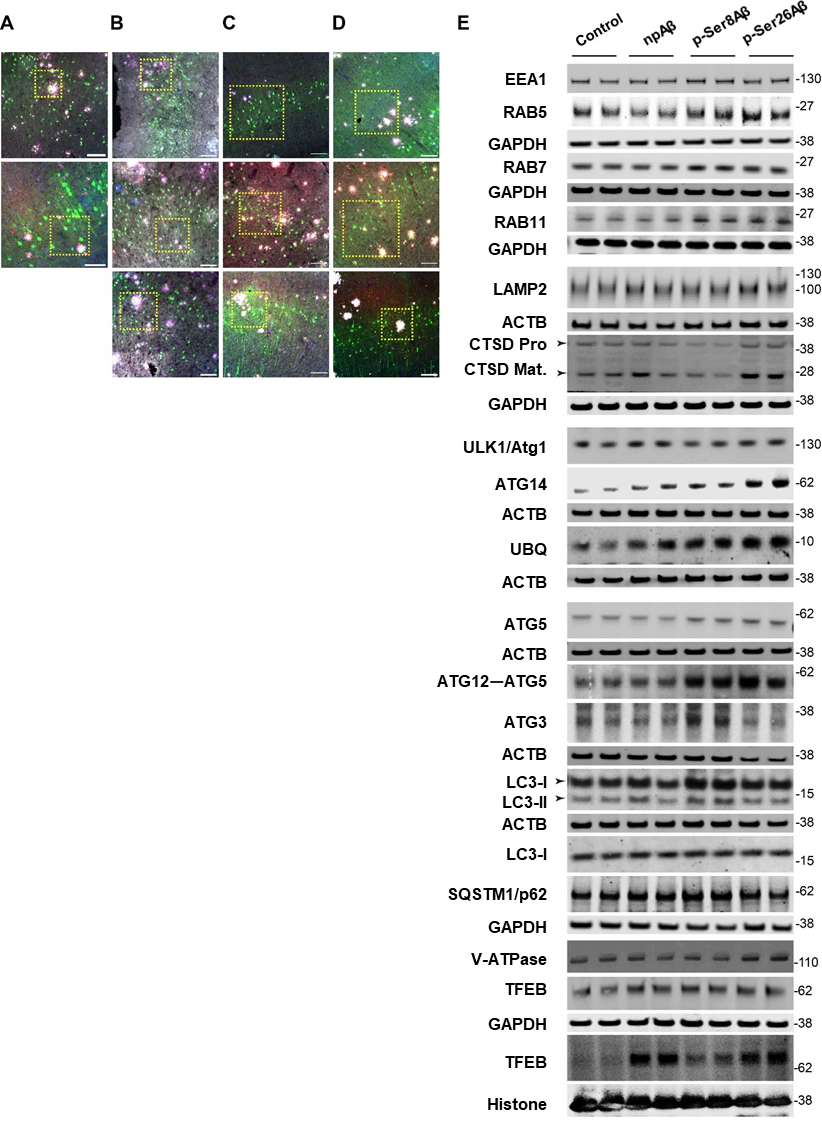
**

**Figure S8.** Source images and representative western immunoblots for the data presented in the paper. (**A-D**) Source images (20x magnification) for Fig. 1A, 2A, 2C, 2E. Scale bar: 100 µm. Region of interest depicted in respective panels have been outlined with yellow dotted box. (**A**) APP-PSEN1dE9xTHY1-YFP transgenic mouse brain sections were stained for p-Ser8Aβ (1E4E11, *red*, **A**-above) p-Ser26Aβ (5H11C10, *red*, **A**-below), and npAβ (7H3D6, *gray*, **A**) along with DAPI + X-34 (nuclei + plaque core, *blue*). (**B-D**) Subsequent brain sections were stained with respective Aβ antibodies (npAβ: 7H3D6; p-Ser8Aβ: 1E4E11 and p-Ser26Aβ: 5H11C10, *red channels respectively*) along with early endosomal marker (EEA1, *red*; **B**); autophagosome marker (LC3, *red*; **C**) and lysosomal marker (LAMP2, *red*; **D**) and DAPI + X-34 (nuclei + plaque core, *blue*). (**E**) Representative WB of different markers used for quantification in Fig. 7E-F and 8B-F.

**Table S1.** Details of the mice analyzed in this study.

| **Figure 1, 2, S1, S2, S8** | | | | |
| --- | --- | --- | --- | --- |
| **Animal**  **(Mouse ID)** | **Genotype** | **Age (months)** | **Sex** | **Background** |
| **1** | THY1-YFP wt/ins | 7.3 | m | C57BL/6 |
| **2** | THY1-YFP wt/ins | 11.4 | f | C57BL/6 |
| **3** | THY1-YFP wt/ins | 13.4 | m | C57BL/6 |
| **4** | THY1-YFP wt/ins | 13.4 | f | C57BL/6 |
| **5** | THY1-YFP wt/ins, APP-PSEN1dE9 wt/tg | 7.4 | m | C57BL/6 |
| **6** | THY1-YFP wt/ins, APP-PSEN1dE9 wt/tg | 7.4 | f | C57BL/6 |
| **7** | THY1-YFP wt/ins, APP-PSEN1dE9 wt/tg | 7.3 | f | C57BL/6 |
| **8** | THY1-YFP wt/ins, APP-PSEN1dE9 wt/tg | 7.3 | f | C57BL/6 |

**Table S2.** Quantification of Aβ levels in sucrose, SDS or formic acid (FA) soluble fractions by ELISA.

| **Aβ species** | **Aβ concentration (pg/µg)** | | | | | |
| --- | --- | --- | --- | --- | --- | --- |
|  | **Sucrose** | | **SDS** | | **FA** | |
|  | **WT** | **APP-PSEN1dE9** | **WT** | **APP-PSEN1dE9** | **WT** | **APP-PSEN1dE9** |
| **Aβ_1-x_** | 0 ± 3.4 | 220.4 ± 61.8 | 0 ± 11 | 517.6 ± 72.3 | 0.8 ± 41.3 | 790.6 ± 93.5 |
| **nmAβ** | 0 ± 2.3 | 28 ± 4.8 | 0 ± 1.6 | 71.4 ± 4.5 | 0.3 ± 5.2 | 124.1 ± 18.2 |
| **p-Ser8Aβ** | 0 ± 6.8 | 85.8 ± 9.2 | 0 ± 2.5 | 162.6 ± 8.4 | 0.6 ± 10.1 | 338 ± 39.3 |
| **p-Ser26Aβ** | 0 ± 5.7 | 64.5 ± 3.3 | 0 ± 0.6 | 42.9 ± 4 | 0.2 ± 2.6 | 69.3 ± 11.2 |

Aβ species in different fractions were captured with either generic anti-Aβ_1-x_ (82E1); anti-nmAβ (7H3D6); phosphorylation state specific antibodies; anti-p-Ser8Aβ (1E4E11) and anti-p-Ser26Aβ (5H11C10), respectively. Bound Aβ was detected with biotin conjugated anti-Aβ (4G8) detection antibody. Raw values were normalized to respective WTxTHY1-YFP mouse brain fractions and values represent mean ± S.D, from four different mouse brains per cohort, from two independent experiments, n = 4 mice, N = 2.

**Table S3.** Levels of different marker proteins for autophagy and the endo-lysosomal pathway analyzed by SDS-PAGE and immunoblotting of membrane and cytosolic* fractions from primary cortical neurons.

|  | **Control** | **npAβ** | **p-Ser8Aβ** | **p-Ser26Aβ** |
| --- | --- | --- | --- | --- |
| **EEA1*** | 1 ± 0.05 | 1.22 ± 0.15 | 1.65 ± 0.21 | 1.28 ± 0.12 |
| **RAB5*** | 1 ± 0.02 | 1.08 ± 0.07 | 1.26 ± 0.09 | 1.47 ± 0.11 |
| **RAB7*** | 1 ± 0.09 | 1.1 ± 0.08 | 0.95 ± 0.17 | 1.25 ± 0.12 |
| **RAB11*** | 1 ± 0.05 | 1.47 ± 0.22 | 1.75 ± 0.29 | 1.89 ± 0.06 |
| **LAMP2** | 1 ± 0.04 | 1.16 ± 0.08 | 0.97 ± 0.06 | 1.23 ± 0.07 |
| **CTSD mature*** | 1 ± 0.03 | 1.08 ± 0.07 | 0.94 ± 0.09 | 1.46 ± 0.11 |
| **CTSD pro*** | 1 ± 0.02 | 0.99 ± 0.06 | 0.99 ± 0.04 | 1.19 ± 0.05 |
| **ATG14** | 1 ± 0.06 | 1.27 ± 0.08 | 1.17 ± 0.05 | 1.58 ± 0.17 |
| **ULK1/Atg1** | 1 ± 0.06 | 1.37 ± 0.35 | 0.88 ± 0.22 | 1.74 ± 0.36 |
| **UBQ*** | 1 ± 0.08 | 1.36 ± 0.19 | 1.62 ± 0.14 | 2.11 ± 0.4 |
| **ATG5** | 1 ± 0.05 | 1.08 ± 0.11 | 1.18 ± 0.1 | 1.31 ± 0.11 |
| **ATG12–­ATG5** | 1 ± 0.04 | 1.04 ± 0.09 | 1.18 ± 0.2 | 1.43 ± 0.23 |
| **ATG3** | 1 ± 0.04 | 1.23 ± 0.21 | 1.23 ± 0.38 | 1.38 ± 0.32 |
| **LC3-I** | 1 ± 0.04 | 1.37 ± 0.06 | 1.43 ± 0.15 | 1.45 ± 0.12 |
| **LC3-II** | 1 ± 0.06 | 1.48 ± 0.06 | 1.95 ± 0.14 | 1.42 ± 0.2 |
| **SQSTM1/p62*** | 1 ± 0.04 | 1.19 ± 0.02 | 1.49 ± 0.06 | 1.41 ± 0.08 |

Primary cortical neurons were treated without (control) or with different Aβ variants (500 nM, 4 h) were subjected to membrane and cytosolic* fractionation. All raw values were normalized to Actin/GAPDH as loading controls and presented as fold change *wrt* control cells, values represent mean ± S.E.M.; n = 6, N = 3.
